# *Aspergillus niger* is a superior expression host for the production of bioactive fungal cyclodepsipeptides

**DOI:** 10.1186/s40694-018-0048-3

**Published:** 2018-03-02

**Authors:** Simon Boecker, Stefan Grätz, Dennis Kerwat, Lutz Adam, David Schirmer, Lennart Richter, Tabea Schütze, Daniel Petras, Roderich D. Süssmuth, Vera Meyer

**Affiliations:** 1 Department Biological Chemistry, Institute of Chemistry, Technische Universität Berlin, Straße des 17. Juni 124, 10623 Berlin, Germany; 2 Department Applied and Molecular Microbiology, Institute of Biotechnology, Technische Universität Berlin, Gustav-Meyer-Allee 25, 13355 Berlin, Germany

**Keywords:** *Aspergillus niger*, Natural products, Non-ribosomal peptide synthetase, Tet-on, Enniatin, Beauvericin, Bassianolide, Precursor directed biosynthesis, Stable isotope dilution analysis

## Abstract

**Background:**

Fungal cyclodepsipeptides (CDPs) are non-ribosomally synthesized peptides produced by a variety of filamentous fungi and are of interest to the pharmaceutical industry due to their anticancer, antimicrobial and anthelmintic bioactivities. However, both chemical synthesis and isolation of CDPs from their natural producers are limited due to high costs and comparatively low yields. These challenges might be overcome by heterologous expression of the respective CDP-synthesizing genes in a suitable fungal host. The well-established industrial fungus *Aspergillus niger* was recently genetically reprogrammed to overproduce the cyclodepsipeptide enniatin B in g/L scale, suggesting that it can generally serve as a high production strain for natural products such as CDPs. In this study, we thus aimed to determine whether other CDPs such as beauvericin and bassianolide can be produced with high titres in *A. niger*, and whether the generated expression strains can be used to synthesize new-to-nature CDP derivatives.

**Results:**

The beauvericin and bassianolide synthetases were expressed under control of the tuneable Tet-on promoter, and titres of about 350–600 mg/L for bassianolide and beauvericin were achieved when using optimized feeding conditions, respectively. These are the highest concentrations ever reported for both compounds, whether isolated from natural or heterologous expression systems. We also show that the newly established Tet-on based expression strains can be used to produce new-to-nature beauvericin derivatives by precursor directed biosynthesis, including the compounds 12-hydroxyvalerate-beauvericin and bromo-beauvericin. By feeding deuterated variants of one of the necessary precursors (d-hydroxyisovalerate), we were able to purify deuterated analogues of beauvericin and bassianolide from the respective *A. niger* expression strains. These deuterated compounds could potentially be used as internal standards in stable isotope dilution analyses to evaluate and quantify fungal spoilage of food and feed products.

**Conclusion:**

In this study, we show that the product portfolio of *A. niger* can be expanded from enniatin to other CDPs such as beauvericin and bassianolide, as well as derivatives thereof. This illustrates the capability of *A. niger* to produce a range of different peptide natural products in titres high enough to become industrially relevant.

**Electronic supplementary material:**

The online version of this article (10.1186/s40694-018-0048-3) contains supplementary material, which is available to authorized users.

## Background

**Fig. 1:**
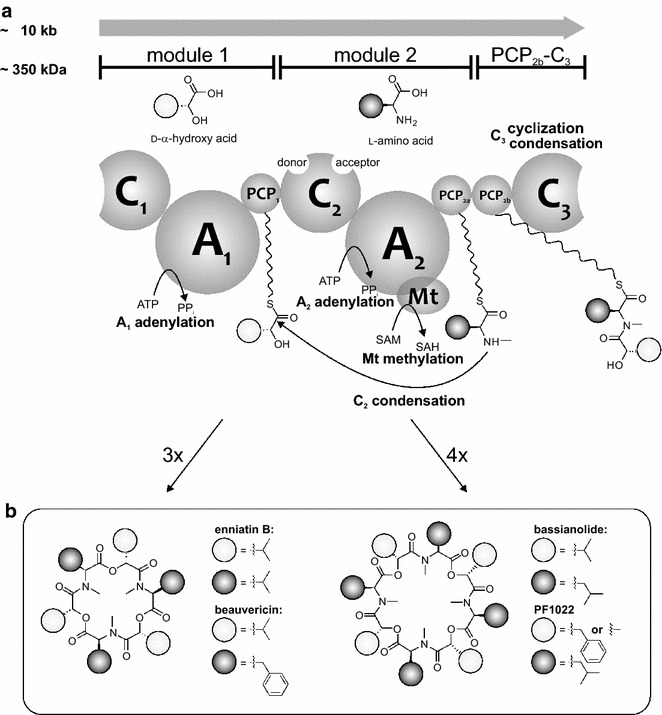
Biosynthesis and structures of fungal CDPs. **a** Proposed biosynthesis of fungal CDPs. The d-hydroxycarboxylic acid is activated by module 1, while the l-amino acid is activated and methylated by module 2. Both substrates are coupled until a hexa- or octapeptidol is formed. Release from the enzyme at the C_3_ domain occurs under cyclization. According to the “parallel” model, elongation of the depsipeptide chain occurs by the addition of dipeptidol units. In the “linear” or “looping” model, single building blocks are attached to the growing depsipeptide chain, which is shuttled between the PCP_1_ and PCP_2a/b_ domains. **b** Structures of enniatin B, beauvericin, bassianolide and PF1022

Fungal cyclodepsipeptides (CDPs), such as enniatins, beauvericins, bassianolide or PF1022 (Fig. 1b), comprise a class of secondary metabolites produced by (mostly pathogenic) filamentous fungi including *Fusarium oxysporum*, *Beauveria bassiana* or *Rosellinia* spp. [1]. They exhibit a variety of different pharmaceutically relevant bioactivities, including antibiotic, anthelmintic, cytotoxic, phytotoxic, insecticidal and anti-retroviral activities [1–4]. Additionally, some fungal CDPs are promising lead structures for new anti-cancer drugs [5–9]. These molecules are symmetric and consist of *N*-methylated l-amino acids and d-hydroxycarboxylic acids, which are alternatingly linked to each other by amide and ester bonds. These CPDs are produced by highly homologous iteratively working bi-modular non-ribosomal peptide synthetases (NRPSs), i.e. enniatin synthetase (ESyn), beauvericin synthetase (BeauvSyn), bassianolide synthetase (BassSyn) and PF1022 synthetase (PFSyn) [1, 2, 10]. Each module is composed of a condensation (C), an adenylation (A), and a peptidyl carrier protein (PCP) domain, whereby module 2 is linked to a terminal PCP_2b_-C_3_ bidomain (Fig. 1a).

At module 1, a d-hydroxycarboxylic acid is activated at the adenylation domain (A_1_) and covalently bound to the peptidyl carrier protein domain (PCP_1_). The l-amino acid is activated at the A-domain of module 2 (A_2_), which is bound to the adjacent PCP_2a_ domain and methylated at the methylation domain (Mt). For substrate elongation, two different models have been proposed. At the “parallel” model, the depsipeptide chain grows by the addition of a dipeptidol, consisting of a hydroxy acid and an *N*-methyl amino acid, previously coupled in the C_2_ domain. In this model, either PCP_2a_ or PCP_2b_ act as a so-called waiting position until the next dipeptidol is formed. Ester bond formation as well as macrocyclization occurs at the C_3_ domain. However, based on recent results by Yu et al. [11] and from our group [12], experimental evidence points to the “linear” or “looping” model: the elongation occurs by the attachment of a single building block (hydroxy acid or *N*-methyl amino acid), while the growing depsipeptide chain is passed between the PCP_1_ and the PCP_2a/b_ domains. Peptide bond formation is catalysed in the C_2_ domain, while the C_3_ domain catalyses ester bond formation and macrocyclization. In this model, the role of the double PCP_2a/b_ domains remains unclear, as either one of the domains is sufficient for biosynthesis of the final product [11, 12]. It was proposed that C_1_ has no direct catalytic function, because truncated CDP synthetases missing the C_1_ domain are still functional. Thus, C_1_ could rather have a stabilizing or supportive role during the catalytic cycle [12]. Currently, it is not clear which of these two models accurately represents NRPs activity, and more investigations are required to fully understand the underlying mechanism of CDP biosynthesis. Due to their high degree of similarity, fungal CDP synthetases are ideal systems for combinatorial biosynthesis approaches, such as module and domain swapping, to obtain novel ‘new-to-nature’ compounds [11–14].

One option for obtaining fungal CDPs is via chemical synthesis, and several such strategies have thus far been described. However, *N*-methylation of amino acids, racemisation during the coupling of hydroxyl acids, as well as the final cyclization step, severely limit the effectiveness of these approaches [2]. For instance, an improved protocol for the total synthesis of enniatin established by Ley and co-workers requires nine steps and results in an overall yield of 15% [15]. Recently, the same group established a protocol based on flow chemistry and were able to synthesize different natural and unnatural CDPs with higher yields (32–52%) [16]. However, high amounts of solvents and costly catalysts make this process uneconomical. A novel chemical synthesis approach using salt additives to support ring formation has been described to synthesize bassianolide, its closely related CDP verticilide [17], and a number of unnatural CDPs with varying ring sizes [18]. Although yields of bassianolide (9%) were almost twice as high as for the first total synthesis published (5.9%) [19], these overall yields are comparably low for production purposes. An alternative and more sustainable way to produce CDPs (and more generally natural products) is by using a biotechnological approach. Here, it is advantageous to transfer the biosynthetic pathway of the natural product of interest from a microbiologically challenging, genetically intractable, or even pathogenic organism into a safe, genetically amendable and industrially established heterologous production host. In the case of CDPs, natural production strains have been established and the highest titres reported for beauvericin production by *Fusarium oxysporum* KFCC 11353P and *Fusarium redolens* Dzf2, which range between 400 and 420 mg/L, respectively [20, 21]. However, not many tools for their genetic modification are available. Production of fungal CDPs in heterologous bacterial hosts has been established, but only low titres were achieved. In the case of beauvericin biosynthesis in *Escherichia coli*, only 8 mg/L were produced [22]. Additionally, enniatin production using *Bacillus subtilis* yielded titres which were also only in the mg/L range [23]. Encouragingly, when *Saccharomyces cerevisiae* was used as heterologous host, higher CDP titres were reported: 74.1 mg/L for beauvericin and 26.7 mg/L for bassianolide [24]. Recently, we were able to show that the industrial fungus *Aspergillus niger*, well-known for its high level production of organic acids and secreted proteins [25], is a promising host for heterologous production of enniatin. In this study, the ESyn encoding gene was put under control of the inducible Tet-on expression system [26] allowing high enniatin titres up to 4.5 g/L upon addition of the inducer doxycycline (Dox) [27]. This strain relies on feeding with the substrate d-hydroxy isovalerate, as it lacks the ketoisovalerate reductase gene *kivR* responsible for the generation of d-Hiv from 2-ketoisovalerate [28]. Autonomous expression strains of *A. niger* independent of d-Hiv feeding were additionally established. In these strains, the *kivR* gene was either monocistronically or polycistronically co-expressed with the ESyn gene [27, 29].

In the present study, we determined whether the *Beauveria bassiana* CDPs beauvericin and bassianolide can also be produced in *A. niger* with high titres. Furthermore, we aimed to test whether the *A. niger* production strains, which lack the ketoisovalerate reductase gene *kivR*, can be used to generate new-to-nature beauvericin derivatives by precursor directed biosynthesis, which ultimately generated CDP variants that are accessible for further downstream chemical modifications.

## Results and discussion

### Generation of *A. niger* strains expressing BeauvSyn and BassSyn

*Aspergillus niger* is an excellent production organism for the synthesis of the hexamer enniatin, which consists of the two building blocks l-valine (l-Val) and d-hydroxy isovalerate (d-Hiv). To show that other CDPs relying on different precursor compositions or different ring sizes can be produced with high titres, our aim was to establish new production strains in an analogous fashion. Therefore, the Tet-on driven expression plasmids pDS8.2 (harbouring *bbBeas* encoding BeauvSyn), and pSB22.3 (harbouring *bbBsls* encoding BassSyn), were constructed and transformed into the *A. niger* strain AB1.13 (see Methods). This isolate is a useful production platform due to reduced protease activities [30]. Transformants carrying a single copy of the expression constructs integrated at the *pyrG* locus were verified by PCR and Southern blot (Additional file 1: Figure S1). Positive strains were cultivated as previously described [27], specifically in 20 mL media in shake flasks, which were then tested for production of the respective CDP. The metabolites were extracted from the dried biomass of the transformants and analysed by LC–MS. The identity of beauvericin and bassianolide was verified by tandem mass spectrometry (Additional file 1: Figures S2 and S3). The relative amounts of produced beauvericin and bassianolide were quantified by multiple reaction monitoring mass spectrometry and the beauvericin-producing strains DSc1.4 (single integration) and DSc1.5 (tandem integration), as well as the bassianolide-producing strain SB19.23 (single integration), which were each selected for further analysis.

### Medium optimization and CDP purification

As indicated above, *A. niger* lacks the *kivR* gene, and consequently feeding of the precursor d-Hiv is necessary. To investigate the impact of precursor concentration on the product titres, d-Hiv as well as the respective amino acid were added to the culture broth in different concentrations. For better comparability, DSc1.4 and SB19.23 were chosen for these studies, as they have a single copy of the respective expression construct integrated at the *pyrG* locus. As shown in Table 1, titres of beauvericin and bassianolide are significantly increased by the addition of the respective precursors. Titres of beauvericin increase from 0.45 ± 0.13 mg/L to 293.62 ± 186.46 mg/L (n = 4) and of bassianolide from 1.04 ± 0.33 mg/L to 378.77 ± 59.74 mg/L (n = 4). Precursor concentrations higher than 15 mM did not further increase the titres (data not shown). Apparently *A. niger* is able to synthesize d-Hiv in small amounts, as both metabolites are formed, even without addition of any precursor. This was also observed for the production of enniatin in *A. niger* [27] and could be due to a relaxed substrate specificity of other endogenous reductases of *A. niger* such as the 2-dehydropantoate 2-reductase (An11g09950), which shows a 28% similarity to KivR from *F. oxysporum* (BLASTP, [31, 32]).

**Table 1:** Titres of beauvericin and bassianolide obtained in shake flask cultivations of *A. niger*

Concentration of amino acid and hydroxy acid precursor (mM)	Titre (mg/L)*	
	Beauvericin	Bassianolide
0	0.45 ± 0.13 (DSc1.4)	1.04 ± 0.33 (SB19.23)
2.5	83.42 ± 5.24 (DSc1.4)	45.90 ± 11.05 (SB19.23)
15	293.62 ± 186.46 (DSc1.4)	378.77 ± 59.74 (SB19.23)
	628.4 ± 211.1 (DSc1.5)	

Titres of beauvericin and bassianolide produced in the transformants DSc1.4, DSc1.5 and SB19.23 with and without precursor addition (d-Hiv and l-Phe for DSc1.4/DSc.15 and d-Hiv and l-Leu for SB19.23)

Respective strains analysed are given in brackets. *: Titres are given in mean ± SD (n = 4 for strains DSc1.4 and SB19.23, n = 5 for strain DSc1.5)

**Fig. 2:**
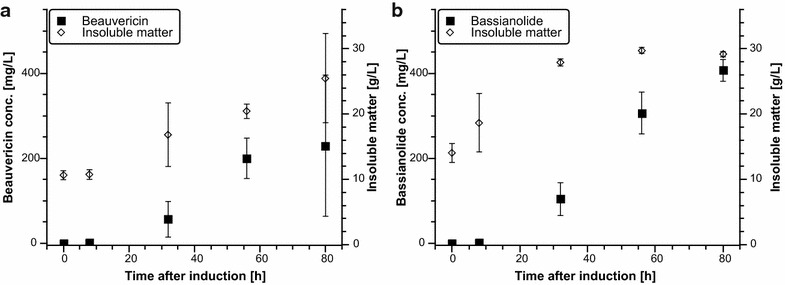
Biomass, beauvericin and bassianolide accumulation over time in shake flask cultivations. **a** Shake flask cultivations of strain DSc1.4. **b** Shake flask cultivations of strain SB19.23. Titres and insoluble matter (micro talc particles and biomass) concentrations are given in means from biological triplicates, SD is indicated by error bars. Note that biomass accumulation in the beauvericin-producing strain DSc1.4 (**a**) is delayed compared to the bassianolide-producing strain SB19.23 (**b**)

To investigate biomass and beauvericin/bassianolide accumulation over time, 20 mL shake flask cultivations of DSc1.4 and SB19.23 were performed with 15 mM addition of the respective precursors as described above. Routinely, micro talc particles were added to the shake flask cultures of *A. niger* in order to obtain a homogeneous macroscopic growth morphology [27]. Samples were taken 0, 8, 32, 56 and 80 h after induction with 20 µg/mL of Dox. Usually, induction of secondary metabolite production in fungi coincides with sporulation [33]; however, a Tet-On based expression approach allows uncoupling of CDP synthesis from natural secondary metabolite kinetics. As shown in Fig. 2, beauvericin and bassianolide were indeed synthesized during the exponential growth phase, but achieve their highest titres during late/post exponential growth phase (Fig. 2). To investigate whether copy number of the expression construct affects the amount of synthesized product, strain DSc1.5 (harbouring a tandem copy of the construct at *pyrG*) was cultivated using the same setting as described above. With 628.4 ± 211.1 mg/L (n = 5), the highest titre of beauvericin was achieved after 80 h of cultivation which is about two-fold higher compared to the single copy strain DSc1.4 (Table 1).

For purification of beauvericin and bassianolide, each 5 × 200 mL shake flask cultivations of strains DSc1.5 and SB19.23 were performed. Gene expression and CDP biosynthesis was induced 16 h post inoculation by the addition of 20 µg/mL Dox and each 15 mM of the respective precursors. Biomass was harvested after an overall cultivation time of 96 h, and beauvericin and bassianolide purified as described in the Methods section. The fractions of the HPLC runs containing only the respective CDP (Additional file 1: Figure S4), were pooled, acetonitrile evaporated, and the residues freeze dried. Overall, 306 mg of beauvericin and 172 mg of bassianolide could be purified from each 1 L culture medium. ^1^H-NMR spectra were recorded for both compounds and verified their purity. The signals obtained (Additional file 1: Figure S7) are in full accordance with data from the literature [7, 34].

### Bioreactor scale production of CDPs

Bioreactor cultivations allow tight control of culture conditions (e.g. temperature, pH, dissolved oxygen), and a better nutrient uptake compared to shake flask cultivations, and are thus better suited to perform highly reproducible fermentations. We thus carried out eight independent bioreactor runs in order to analyse the performance and productivity of the single copy beauvericin-producing strain DSc1.4 and the single copy bassianolide-producing strain SB19.23 in more detail.

**Fig. 3:**
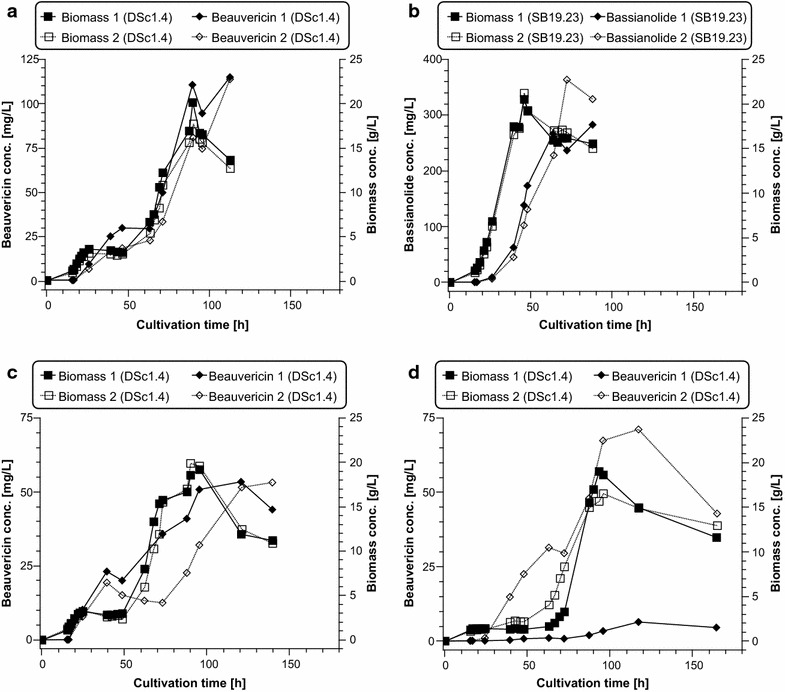
Biomass, beauvericin and bassianolide accumulation over time in bioreactor cultivations. **a** Biomass and beauvericin titres produced by DSc1.4 using the standard cultivation condition (biological duplicate). **b** Biomass and bassianolide titres produced by SB19.23 using the standard cultivation condition (biological duplicate). **c** Biomass and beauvericin titres produced by DSc1.4 when d-Hiv and l-Phe were added prior to inoculation (biological duplicate). **d** Biomass and beauvericin titres produced by DSc1.4 under uncontrolled pH conditions (biological duplicate). Note that different cultivation times are due to the delayed growth of DSc1.4 and were adjusted within the course of this study. Closed symbols: replicate 1; open symbols: replicate 2. For clarity, the symbols have been connected by solid (dashed) lines

For the first set of fermentations, expression was induced with 20 µg/mL of Dox and the respective precursors (amino acid and hydroxy acid) were each added to a final concentration of 15 mM 16 h post inoculation (standard cultivation conditions).The two DSc1.4 bioreactor experiments show an unusual growth behaviour compared to strain SB19.23 (Fig. 3a, b). DSc1.4 stops growing after approximately 24 h post inoculation for about 36 h and resumes growth after nearly 60 h until it reaches its maximum biomass concentration (20.0 and 17.6 g/L) after 90 h of cultivation. Such a growth profile is reminiscent of a diauxic shift, a known growth phenomenon that occurs when different C- or N-sources are simultaneously present in a medium [35]. In contrast, strain SB19.23 grows continuously as expected with maximal growth rates of µ_max_ = 0.178 and 0.174 h^−1^, respectively, until it reaches its maximum biomass concentration (20.5 and 21.2 g/L) after 46 h. Notably, DSc1.4 also showed a delayed growth behaviour during shake flask cultivations (Fig. 2). Most interestingly, the bassianolide titres of strain SB19.23 obtained from bioreactor runs are in the same range as measured for shake flask cultivations (~ 300 mg/L), whereas the beauvericin titres of DSc1.4 obtained in bioreactor runs (~ 100 mg/L) were 3–4 fold lower compared to shake flask cultivations. We speculated that the diauxic shift observed in DSc1.4 cultivations might be causatively linked to the addition of l-Phe to the medium 16 h post inoculation. l-Phe is known to serve as an alternative nitrogen as well as carbon source to fungi [36]. When d-Hiv and l-Phe were added at the beginning of the bioreactor cultivations, the diauxic growth curve was still observed, and beauvericin titres even dropped to 50 mg/L (Fig. 3c).

To further investigate the phenomenon of decreased beauvericin titres obtained from bioreactor runs compared to shake flask cultivations, a third set of experiments was performed. Another parameter that is different between shake flask and bioreactor cultivations is ambient pH: The pH of the medium used in shake flask cultivations is set to pH 5.6 and uncontrolled during cultivation, whereas the pH of the fermentation medium in bioreactor cultivations is adjusted to pH 3.0 and kept constant during fermentation. Thus, the pH of the medium used for the final two bioreactor runs was set to pH 5.6 and the pH control system switched off. Precursors were added at the same time point as in the shake flask cultivations (after 16 h). The growth behaviour of DSc1.4 still followed a pronounced diauxic shift pattern and beauvericin titres were again lower in comparison to shake flask cultivations (Fig. 3d). Also, the overall growth of the strain in these two runs was slower (especially of replicate 1) compared to the previous runs (Fig. 3a, c) and the beauvericin titre was, with 6.3 mg/L for replicate 1, the lowest of all runs (Fig. 3d). From this experiment, we concluded that a controlled, low ambient pH is in favour of growth of *A. niger* and thus (because Tet-on driven expression couples CDP synthesis with growth) high beauvericin titres. Additionally, growth as well as beauvericin production showed a high deviation under uncontrolled pH conditions. An overview of all titres obtained from bioreactor cultivations is summarised in Table 2.

**Table 2:** Titres of beauvericin and bassianolide obtained from bioreactor cultivations

Cultivation condition	Maximum titre (mg/L)	
	Beauvericin (run 1/run 2)	Bassianolide (run 1/run 2)
Standard cultivation condition	113.9/112.9	281.8/362.5
Precursor addition after 0 h	53.1/52.9	n.d.
No pH control	70.7/6.3	n.d.

Titres of beauvericin and bassianolide produced in transformants DSc1.4 and SB19.23 under standard and modified cultivation conditions (for details see text). Bioreactor runs were performed in duplicates (run 1 and run 2)

*n.d.* not done

**Fig. 4:**
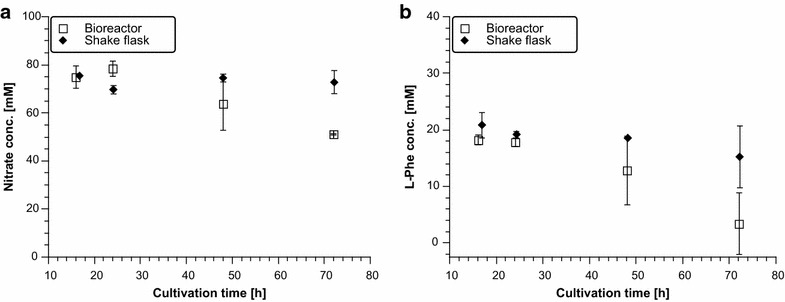
Nitrate and l-Phe concentrations obtained from shake flask and bioreactor cultivations of strain DSc1.4. **a** Nitrate concentration in medium of shake flask and bioreactor cultivations; **b** l-Phe concentration in medium of shake flask and bioreactor cultivations. Measurements for bioreactor runs were done in biological duplicates, for shake flask cultivations in biological triplicates

While all medium parameters (except pH) were comparable in shake flask and bioreactor cultivations, oxygen supply differed considerably. In bioreactor cultivations, *A. niger* is constantly supplied with 2 L air/min while *A. niger* likely encounters hypoxic conditions in shake flask cultivations, as the cotton plugs do not guarantee optimal gas transfer into the medium [37]. The nitrogen source in the medium used for both bioreactor and shake flask cultivations is nitrate. Nitrate is known as a secondary nitrogen source: under normoxic conditions, it is reduced to nitrite and ammonium before assimilated into the biomass [38], whereas under hypoxic conditions, it can additionally serve as a terminal electron acceptor during energy generation, a process called nitrate respiration [39]. As under normoxic conditions nitrate needs first to be reduced to ammonium, it could be energetically more feasible for *A. niger* to use l-Phe as nitrogen source, as shown for the model fungus *Neurospora crassa* [40]. Thus, more l-Phe could be used for biomass production rather than beauvericin biosynthesis, which would offer an explanation for reduced beauvericin titres during bioreactor cultivations. In any case, one would hypothesize that both nitrate and l-Phe are consumed faster during shake flask cultivations in comparison to bioreactor cultivations. This is what we could indeed observe (Fig. 4). From the data obtained, it cannot be deduced how much l-Phe is used for beauvericin synthesis and how much for biomass accumulation. Clearly, most of the added l-Phe is used for biomass accumulation in both cultivation conditions, as the l-Phe concentration decreased from 18.2 to 3.4 mM in shake flasks and from 20.8 to 15.2 mM in bioreactors, which would correspond to theoretical beauvericin titres of 3.86 g/L in shake flasks and 1.47 g/L in bioreactors respectively. Recently, Kniemeyer and co-workers studied the importance of oxygen on secondary metabolism in *A. fumigatus*. They showed that the production of some secondary metabolites depends on both the developmental stage of the fungus and on the available oxygen level [41–43]. The PKS/NRPS product pseurotin A, for example, is strongly up-regulated under hypoxia [41]. In the Tet-on based *A. niger* expression strains used in this study, transcription of the BeauvSyn is likely not affected by the concentration of dissolved oxygen in the medium, however, expression levels of other enzymes involved in the synthesis of beauvericin [e.g. phosphopantetheinyl transferases, CoA synthesis, *S*-adenosyl methionine synthesis] could be positively linked to a lower oxygen supply in shake flask cultures. Interestingly, many genes involved in l-Phe biosynthesis of *A. fumigatus* are higher expressed under hypoxic in comparison to normoxic conditions [43]. Also genes involved in the metabolism of secondary products derived from l-phenylalanine, and in the *S*-adenosyl-methionine-homocystein cycle were up-regulated under hypoxic conditions [43]. Overall, these observations support the hypothesis that higher beauvericin titres in shake flask cultivations of *A. niger* might be mechanistically linked with reduced oxygen levels and thus altered l-Phe metabolism. Further analysis to prove/disprove this speculation goes beyond the scope of this article; however, it clearly demonstrates that further optimization studies regarding beauvericin overproduction in *A. niger* should also consider metabolic engineering aspects that address precursor availability and consumption.

### Generation of non-natural CDP derivatives

We next tested whether the beauvericin and bassianolide producing *A. niger* strains DSc1.4 and SB19.23 can be used to produce new-to-nature beauvericin and bassianolide derivatives by exploiting the relaxed substrate specificity of A_1_ domains towards d-Hiv [44–46]. In non-autonomous production strains, substrate analogues do not compete with the natural substrates, and a precursor directed biosynthesis approach can therefore be applied [47, 48]. This technique, also called mutasynthesis or mutational biosynthesis, has been previously applied to obtain non-natural beauvericins from the heterologous host *E. coli*, as well as from a *kivR* deletion mutant of the natural producer *B. bassiana*. However, the titres for most of the non-natural beauvericin analogues stayed in the low mg/L range and only beauvericin analogues could be isolated when the alternative hydroxy acid displayed similar properties as d-Hiv (aliphatic side chains) [45, 47].

**Fig. 5:**
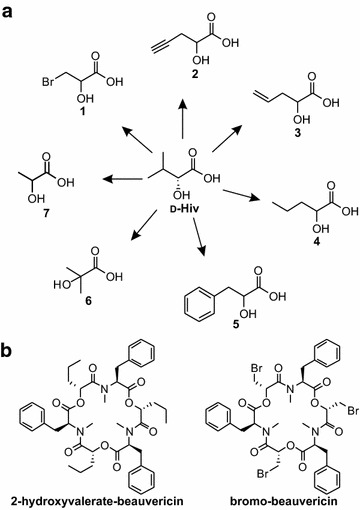
Hydroxy acids fed to strains DSc1.4 and SB19.23 in order to synthesize new-to-nature beauvericins. **a** Seven artificial hydroxy acid analogues (1–7) were fed to strains DSc1.4 and SB19.23, varying in the length of the side chain (4, 6, 7), bearing a halogen (1), aromatic (5), alkene (3) or alkyne (2) functionality. Final precursor concentration was 7.5 mM. **b** Non-natural beauvericins (2-hydroxyvalerate-beauvericin and bromo-beauvericin) which were successfully produced by strain DSc1.4

Here, we tested seven different d-Hiv analogues, which were fed to strains DSc1.4 and SB19.23 in shake flask cultivations in final concentrations of 7.5 mM. We decided to use lower feeding concentrations of hydroxy acids to decrease the risk of toxic effects of the precursors on *A. niger*. As a consequence, the corresponding amino acid was also added at 7.5 mM to the cultures. The strains were cultivated in 20 mL scale as described for the natural beauvericin and bassianolide derivatives, the biomass extracted, and the extracts analysed by LC–MS. From all tested compounds, only 2-hydroxyvalerate and 3-bromo-lactate were incorporated by DSc1.4 into the beauvericin backbone, leading to the novel beauvericin analogues 2-hydroxyvalerate-beauvericin and bromo-beauvericin (Fig. 5b and Additional file 1: Figure S8). Interestingly, a beauvericin derivative with incorporated propargyl-lactate could not be detected, although the hydroxy acid had been accepted in in vitro and in vivo experiments in *E. coli* [45]. This could be due to a metabolization of the acid by *A. niger* as also observed in *Corynebacterium glutamicum* [49]. Surprisingly, SB19.23 was not able to synthesize any artificial bassianolide derivative, suggesting that the substrate specificity of the BassSyn is tightly controlled compared to BeauvSyn. Alternatively one may assume that the altered side chains with their sterically more demanding substituents interfere with ring formation of an cyclooctadepsipeptide.

Because feeding of bromo-lactate resulted in significant amounts of bromo-beauvericin, we wanted to assess the capacity of this approach for production and isolation on a larger scale. As a preliminary experiment, different concentrations of the racemic precursor d/l-bromo-lactate (5, 10 and 15 mM) were added to DSc1.4 in small scale cultivations (20 mL scale). The addition of 10 mM of d/l-bromo-lactate gave higher titres than the addition of 5 mM, while no bromo-beauvericin could be detected in the cultures supplemented with 15 mM of the hydroxy acid. This coincided with significant less biomass formation, suggesting that high concentrations of bromo-lactate have toxic effects on *A. niger*. Based on these results, a concentration of 10 mM of d/l-bromolactate was chosen for large scale cultivation in shake flasks. 12 mg of pure bromo-beauvericin (preparative HPLC) could be successfully obtained as a colourless powder from a 1.1 L of culture of DSc1.4, the purity of which was proven by mass spectrometry and NMR (Additional file 1: Figures S9 and S10). The purified bromo-beauvericin was tested for antimicrobial and antiparasitic activity together with purified enniatin B, beauvericin and bassianolide. While bromo-beauvericin did not show any improved antimicrobial or antiparasitic activity compared to the other compounds, it interestingly did not show any cytotoxic effects against a mammalian cell line at a concentration of 100 µg/mL, whereas the IC_50_ value of natural beauvericin is 1.52 µg/mL. It is thus worth studying bromo-beauvericin further as a potential future antiparasitic drug (Additional file 1: Table S4).

### Generation of deuterated CDP standards

Fungal CDPs are not only of pharmaceutical interest as lead structures, but are also prominent contaminants (especially enniatins and beauvericin) of food and feed, as most of their natural producers are plant pathogenic fungi [50–52]. Thus, robust, fast, and exact analytical methods are needed to detect and quantify these compounds, even in trace amounts, in both food and feed products suspected to be spoiled by fungi. Most described protocols are based on LC–MS measurements in combination with an external standard calibration curve [53–56]. However, these methods are only exact to a certain degree as they do not consider effects of the matrix which can lead to ion suppression or ion enhancement [57, 58]. Furthermore, the recovery rates of the analytes from biological samples may vary, which would also lead to altered results [53]. Stable isotope dilution assays are superior to methods using external standards as they guarantee exact quantifications also of mycotoxins in grain products [58–61]. The biosynthesis of ^15^N_3_-labelled standards of enniatins and beauvericin in *F.* *sambucinum* (enniatin producer) and *F. fujikuroi* (beauvericin producer) grown on Na^15^NO_3_ as sole nitrogen source has been reported [62]. With 430 µg (enniatin A), 450 µg (enniatin A1), and 1460 µg (beauvericin) of ^15^N-labelled compound purified from 500 mL of culture, titres are low while the price of the medium is relatively high.

**Fig. 6:**
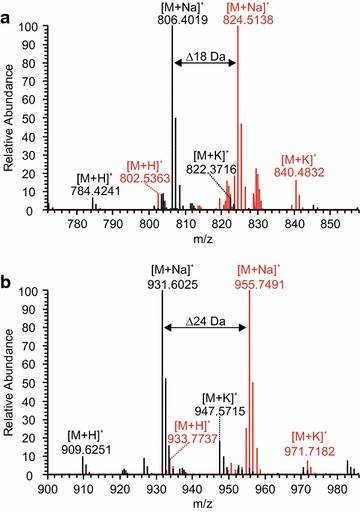
Mass shifts observed for deuterium-labelled beauvericin and bassianolide. Overlaid mass spectra of purified deuterium-labelled (red) and unlabelled (black) beauvericin (**a**) and bassianolide (**b**). Because three molecules of d-Hiv-d_6_ are incorporated into beauvericin and four into bassianolide, mass shifts of 18 and 24 Da can be expected and have indeed been observed

We thus tested the transformants DSc1.4 and SB19.23 for their suitability to synthesize labelled beauvericin and bassianolide. Instead of ^15^N, we used deuterium (^2^H) to label d-Hiv. d/l-Hiv-d_6_ was synthesized using acetone-d_6_ as starting material. The respective expression strains were cultivated in shake flasks (100 mL scale) and 15 mM of d/l-Hiv-d_6_ together with 7.5 mM of the respective l-amino acid were added. Because a racemic mixture of d/l-Hiv-d_6_ was fed to the cultures, lower titres of beauvericin and bassianolide were expected compared to the purification of unlabelled metabolites. Cultivation and purification of both compounds were carried out as described above. From 100 mL of culture, 8.2 mg of beauvericin-d_18_ and 5.7 mg of bassionalide-d_24_ were isolated in analytically pure form (Fig. 6). Retention times between natural and deuterated variants of beauvericin and bassianolide differed slightly on a C18 reverse phase column. This effect has also been described earlier and is due to subtle differences in the polarity of labelled and unlabelled compounds (Additional file 1: Figure S6) [63].

Because sample preparation for LC–ESI–MS analysis from complex matrices (e.g. fungal biomass or grain products) can be laborious and time-consuming, especially if many samples need to be tested, it was evaluated whether the deuterated beauvericin and bassianolide standards could also be used for quantification on a MALDI-TOF instrument. In contrast to LC–ESI–MS, where the samples have to be pre-purified in order to keep the ion source clean, crude extracts can be directly applied to MALDI-TOF. To test this, defined amounts of deuterated beauvericin and bassianolide were added to a dilution series of the unlabelled compounds (Additional file 1: Table S5). The ratio of the peak areas of the sodium adducts (most abundant peaks) of the respective labelled and unlabelled compounds were plotted against the ratio of the concentration of the compounds to determine if a linear relation was observed. The ratios of the concentrations and the peak areas indeed show a linear correlation (Additional file 1: Figure S11). However, as pointed out by the coefficients of determination (*R*^2^ = 0.946 for the beauvericin measurements and *R*^2^ = 0.988 for the bassianolide measurements), quantification of both compounds is not exact. One problem of the MALDI-TOF measurements is that only a direct MS is being recorded and that MS/MS of labelled and unlabelled compound cannot be concomitantly measured. Thus, any compound showing the same or a very similar m/z value to the labelled or unlabelled analytes would alter the results. Nevertheless, the MALDI-TOF measurement is an interesting alternative for high-throughput applications where an approximate estimation of CDP concentrations can be tolerated (e.g. for screening many samples or strains). For exact measurements however, LC–MS/MS remains the method of choice as reviewed in [61].

## Conclusions

Cyclodepsipeptides are potential new drugs for various medicinal applications. Enniatin and beauvericin are discussed as anticancer drugs [8, 9], the PF1022A derivative emodepside is currently in phase I clinical trials and tested as an anthelmintic drug for use in humans [64]. Hence, sustainable approaches to synthesize these compounds at a large scale and in a commercially viable way have become a recent focus in biotechnology. In this and our previous study [27], we have shown that *A. niger* is an excellent expression host for CDPs, including enniatin, beauvericin and bassianolide. The CDP titres reached are, to our knowledge, the highest ever reported for heterologous producers, as well as for natural producing organisms, and are summarised in Table 3. The fact that *A. niger* tolerates and accepts unnatural building blocks makes it furthermore an attractive platform for the production of new-to-nature CDPs by mutasynthesis, as we have demonstrated for the beauvericin derivative bromo-beauvericin. The amounts isolated were indeed high enough for bioactivity testing, which showed comparable antiparasitic activity and, interestingly, markedly reduced cytotoxicity. We have also demonstrated that *A. niger* can be genetically trimmed to synthesize deuterated CDPs which can be exploited as internal standards to evaluate mycotoxin burden of food by stable-isotope dilution assays.

**Table 3:** Summary of highest CDP titres ever reported for bacterial and fungal expression hosts

CDP	Native producer	Heterologous producer	
Enniatin B	5 g/L (enniatin B and other derivatives) *F. oxysporum* Shake flask cultivation [70]	1.1 mg/L *B. subtilis* Shake flask cultivation [23]	950 mg/L *A. niger* Shake flask cultivation [27]
			4.5 g/L *A. niger* Bioreactor cultivation [27]
Beauvericin	420 mg/L *F. oxysporum* Shake flask cultivation [20]	74.1 ± 0.3 mg/L *S. cerevisiae* Shake flask cultivation [24]	628.4 ± 211.1 mg/L *A. niger* Shake flask cultivation [This study]
	22.3 ± 1.5 mg/L *B. bassiana* Shake flask cultivation [24]	8 mg/L *E. coli* Shake flask cultivation [22]	113.4 mg/L *A. niger* Bioreactor cultivation [This study]
Bassianolide	18.2 ± 0.6 mg/L *B. bassiana* In shake flask cultivation [24]	26.7 ± 2.8 mg/L *S. cerevisiae* Shake flask cultivation [24]	378.77 ± 59.74 mg/L *A. niger* Shake flask cultivation [This study]
			322.12 mg/L *A. niger* Bioreactor cultivation [This study]

For simplicity, respective strain names, genetic modifications and details on cultivation conditions (medium composition, cultivation time, feeding conditions, etc.) are not indicated for the different cell factories. This data can be extracted from the references given

## Methods

### Strains and general cloning procedures

Plasmids, primers and strains used in this study are summarized in Additional file 1: Tables S1–S3. Molecular techniques for *E. coli* followed protocols described earlier [65]. *A. niger* transformation and genomic DNA extraction from selected transformants was done according to [66]. The BeauvSyn and BassSyn encoding genes *bbBeas* (GenBank accession number EU886196) and *bbBsls* (GenBank accession number FJ439897) were amplified from the genomic DNA of *B. bassiana* ATCC 7159 using the primer pairs Beauv_InFusion1_fw/Beauv_InFusion3_rv for *bbBeas* and Bass_InFusion1_fw/Bass_InFusion3_rv for *bbBsls*, respectively. The amplicons were ligated into the cloning vector pJET2.1 (Thermo Fisher Scientific Inc.), resulting in pDS2.1 (harbouring *bbBeas*) and pDS1.9 (harbouring *bbBsls*) and verified by restriction analysis and sequencing. Direct cloning of the full-length genes into the *A. niger* Tet-on expression vector pVG2.2 was not successful. Thus, the genes were split into three parts of approximately 3 kbp length and 15 bp overhangs to each other and the *Pme*I-linearized vector pVG2.2. pDS2.1 and pSB1.9 were used as templates and primer pairs Beauv_InFusion1_fw/Beauv_InFusion1_rv, Beauv_InFusion2_fw/Beauv_InFusion2_rv, Beauv_InFusion3_fw/Beauv_In-Fusion3_rv for *bbBeas* and Bass_InFusion1_fw/Bass_InFusion1_rv, Bass_In-Fusion2_fw/Bass_InFusion2_rv, Bass_InFusion3_fw/Bass_InFusion3_rv for *bbBsls* to amplify the respective gene fragments. The amplicons were ligated and assembled into the *Pme*I-linearized Tet-on expression plasmid pVG2.2 via the In-Fusion^®^ HD Cloning Kit (Clontech), resulting in plasmids pDS8.2 (harbouring *bbBeas*) and pSB22.3 (harbouring *bbBsls*).

### Shake flask cultivations of *A. niger*

For production of CDPs, transformants were cultivated in 20 mL or 200 mL enniatin production medium (EM) as described in [27] if not indicated otherwise. Hydroxy and amino acid precursors were added in the range of 0–25 mM. Cultures were inoculated with 5 × 10^6^ spores/mL and Tet-On driven expression induced with 20 µg/mL doxycycline 16 h after inoculation.

### Bioreactor cultivations of *A. niger*

Submerged cultivations were performed with Biostat bioreactors (Sartorius, Göttingen, Germany, 4 L working volume) as described before [27]. Glucose-limited batch cultivation was initiated by inoculation of fermentation medium (CM with 5% of glucose: 7 mM KCl, 11 mM KH_2_PO_4_, 70 mM NaNO_3_, 2 mM MgSO_4_, 1x trace element solution [67], 0.1% casamino acids, 0.5% yeast extract, 5% glucose) with conidial suspension of *A. niger* transformants to give 10^9^ conidia L^−1^. Glucose was sterilized separately from the fermentation medium. Temperature of 26 °C and pH 3 were kept constant if not stated otherwise, the latter by computer controlled addition of 2 M NaOH or 1 M HCl. Computer-controlled base addition to the culture broth was used as an indirect growth measurement [68]. When the culture reached the early exponential growth phase (about 16 h after inoculation, corresponds to 1 g biomass dry weight kg^−1^), Dox (20 μg/ml), d-Hiv (15 mM) and l-Phe or l-Leu (15 mM) were added.

### Purification of CDPs

Purification of CDPs from *A. niger* biomass was adapted from [27]. In brief, the mycelium from a 1 L culture was harvested by suction filtration and lyophilized. The dried mycelium was ground in a mortar and extracted three times with 300 mL of EtOAc. The solvent was evaporated and the brownish residue filtered over a short silica column (*n*-hexanes/EtOAc = 50:50). The solvents were evaporated and the residues resolved in methanol. Insoluble residues were removed by filtration and the solvent evaporated. For beauvericin and bassianolide purification, the residues were dissolved in acetonitrile/water (80:20) and the solution was centrifuged at 10,000×*g* for 15 min to remove insoluble particles. The supernatant was subjected to reversed phase chromatography using a GROM-Sil 120 ODS-5 HE (10 µm, 250 × 20 mm) column on an Agilent 1100 series preparative HPLC system running isocratically on acetonitrile (+ 0.1% formic acid)/water (+ 0.1% formic acid) (70:30) for beauvericin or with a linear gradient (70–100% acetonitrile over 15 min) for bassianolide with a flow rate of 15 mL/min. For bromo-beauvericin purification, the residues were resolved in MeOH and subjected to reversed phase chromatography using a GROM-Sil 120 ODS-5 HE (10 µm, 250 × 20 mm) column on an Agilent 1100 series preparative HPLC system running isocratically on MeOH (+ 0.1% formic acid)/water (+ 0.1% formic acid) (81.5/18.5) with a flow rate of 15 mL/min. Fractions containing the respective CDP were pooled, acetonitrile and MeOH were evaporated and water was removed by freeze drying.

### Analysis and quantification of produced CDPs

Biomass (which included in the case of shake flask cultivations also insoluble parts, i.e. talc particles) of a defined amount of culture broth was harvested by suction filtration and lyophilized and weighed. The biomass was ground and 25 mg were transferred to a 2 mL test tube and extracted with 1 mL of EtOAc, shaking overnight. The tubes were centrifuged at 13,000×*g* and 700 µL of the extract were transferred to a new 1.5 mL test tube and evaporated. The residues were dissolved in 1 mL of water/isopropanol (50:50), diluted if necessary and the amount of CDPs quantified in MRM mode on an ESI-Triple-Quadrupol-MS 6460 Series (Agilent Technologies) coupled to an Agilent 1290 Infinity HPLC system (Agilent Technologies) equipped with an Agilent Poroshell 120 EC-C18 (3.0 × 50 mm) column (Agilent Technologies), heated to 50 °C. The mobile phases were H_2_O (A) and isopropanol (B). The injection volume was set to 3 µl and the flow rate was 0.4 ml/min. The applied gradient was: 50–100% (0.0–3.2 min), 100% (3.2–4.5 min), 100–5% (4.5–4.6 min), 5% (4.6–5.6 min), 5–50% (5.6–5.7 min), 50% B (5.7–7.0 min). For beauvericin quantification, the *m/z* value for the precursor ion was set to 806.4 ([M + Na]^+^ adduct) and for the fragment ion to 384.1 as quantifier, for bassianolide quantification, the *m/z* value for the precursor ion was set to 931.6 ([M + Na]^+^ adduct) and for the fragment ion to 350.1 as quantifier. For every set of measurements, a new calibration curve was made using beauvericin or bassianolide isolated from *A. niger* transformants as an external standard. Peak areas were determined by manual integration using MassHunter Workstation Qualitative Analysis (Agilent Technologies). Exact masses of purified CDPs were recorded on an ESI-LTQ-Orbitrap-MS, Orbitrap XL (Thermo Fisher Scientific). Samples were dissolved in MeOH and measured by direct injection. Analysis was performed with the Xcalibur 2.2 software (Thermo Fisher Scientific). Retention times of labelled and unlabelled beauvericin and bassianolide were determined on an ESI-Orbitrap-MS, Exactive (Thermo Fisher Scientific) coupled to an Agilent 1260 Infinity HPLC system (Agilent Technologies) equipped with an Agilent Poroshell 120 EC-C18 (2.1 × 50 mm) column (Agilent Technologies). The mobile phases were H_2_O + 0.1% formic acid (A) and acetonitrile + 0.1% formic acid (B). The injection volume was set to 2 µl and the flow rate was 0.4 ml/min. The applied gradient was: 40% (0.0–0.5 min), 40–100% (0.5–12.0 min), 100% (12.0–13.5 min), 100–40% (13.5–13.6 min), 40% B (13.6–15.5 min). 1:1 mixtures of labelled and unlabelled beauvericin and bassianolide were injected and retention times of the sodium adducts of each compound determined. Analysis was performed with the Xcalibur 2.2 software (Thermo Fisher Scientific).

For MALDI-TOF analysis, 1 µL of purified CDPs or crude extracts of *A. niger* transformants, solved in MeOH or acetonitrile, were either mixed with 1 µL of saturated 2,5-dihydroxybenzoic acid (DHB) or α-cyano-4-hydroxycinnamic acid (CHCA) solution [dissolved in an acetonitrile–water mixture (1:1), acidified with formic acid (1%)]. 1 µL of the mixture was spotted onto a ground steel MALDI target plate and allowed to dry and crystallize. Measurements were carried out on a Bruker ultrafleXtreme MALDI-TOF–MS, equipped with a smartbeam II laser. The intensity of the laser was set to 50% with a frequency of 2 kHz. Calibration was done with the peptide calibration standard (Bruker). Analysis was performed with the Compass for flexSeries 1.4 software (Bruker).

For NMR analysis, purified CDPs were solved in CDCl_3_ or MeOH-d_4_ and ^1^H-NMR and ^13^C-NMR spectra were recorded on a Bruker Avance III 700 MHz NMR spectrometer or Bruker Avance II 400 MHz NMR spectrometer. The signals of the non-deuterated solvent rests were used as standards. Chemical shifts are given in δ-units (ppm) relative to the solvent signal.

### Synthesis of α-hydroxy acid precursors

Synthesis of d-Hiv and α-hydroxy acid analogues (Fig. 5) followed the procedures described in [46]. The synthesis of d/l-Hiv-d_6_ followed the procedure described in [69].

### Quantification of nitrate and l-Phe

Quantification of nitrate and l-Phe concentrations in the cultivation medium was performed with the Nitrate/Nitrite Colorimetric Assay Kit (Cayman Chemical) and the Phenylalanine Assay Kit (Sigma-Aldrich) according to the manufacturers’ protocols.

### Antimicrobial and antiparasitic test assays

Bioactivity assays were performed as described in [12].

## Additional file

**Additional file 1.** Supplemental Information.
