# Correction to: *Aspergillus niger* is a superior expression host for the production of bioactive fungal cyclodepsipeptides

**DOI:** 10.1186/s40694-018-0051-8

**Published:** 2018-03-27

**Authors:** Simon Boecker, Stefan Grätz, Dennis Kerwat, Lutz Adam, David Schirmer, Lennart Richter, Tabea Schütze, Daniel Petras, Roderich D. Süssmuth, Vera Meyer

**Affiliations:** 1 Department Biological Chemistry, Institute of Chemistry, Technische Universität Berlin, Straße des 17. Juni 124, 10623 Berlin, Germany; 2 Department Applied and Molecular Microbiology, Institute of Biotechnology, Technische Universität Berlin, Gustav-Meyer-Allee 25, 13355 Berlin, Germany

## Correction to: Fungal Biol Biotechnol (2018) 5:4 10.1186/s40694-018-0048-3

Following publication of the original article [1], the authors reported that there is a mistake in the legend of Fig. 4; the explanation of the symbols has been mixed up. The corrected version of Fig. 4 is given below.

**Fig. 4:**
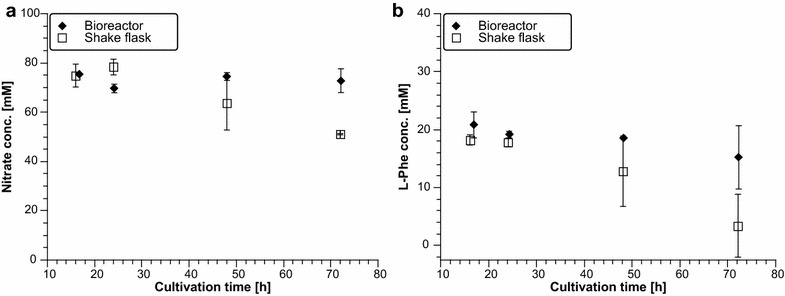
Nitrate and l-Phe concentrations obtained from shake flask and bioreactor cultivations of strain DSc1.4. **a** Nitrate concentration in medium of shake flask and bioreactor cultivations; **b** l-Phe concentration in medium of shake flask and bioreactor cultivations. Measurements for bioreactor runs were done in biological duplicates, for shake flask cultivations in biological triplicates
