# Supplementary material for: Aspergillus niger is a superior expression host for the production of bioactive fungal cyclodepsipeptides
Source: Fungal Biol Biotechnol. 2018 Mar 2;5:4. doi: 10.1186/s40694-018-0048-3 (PMC5833056; doi:10.1186/s40694-018-0048-3)
Supplement: Supplementary file 1 — Additional file 1. Supplemental Information. [file 40694_2018_48_MOESM1_ESM.docx]

**Additional file**

Table S 1 - Plasmids used in this study

| **Name** | **Information** | **Source** |
| --- | --- | --- |
| **pVG2.2** | Tet-on expression vector | [1] |
| **pJET2.1** | Cloning vector | Thermo Fisher Scientific Inc., Waltham (USA) |
| **pDS2.1** | pJET2.1 harbouring *bbBeas* gene | This study |
| **pDS1.9** | pJET2.1 harbouring *bbBsls* gene | This study |
| **pDS8.2** | pVG2.2 harbouring *bbBeas* gene | This study |
| **pSB22.3** | pVG2.2 harbouring *bbBsls* gene | This study |

Table S 2 - Primers used in this study

| **Name** | **Sequence (5' - 3')** |
| --- | --- |
| **Beauv_InFusion1_fw** | CAGACATCACCGTTTACCATGGAGCCGCTCAAAAATGTCAATAC |
| **Beauv_InFusion1_rev** | CTTGAGAGTAAGACTGCTCAACGGGTCCGTCCCAAGTC |
| **Beauv_InFusion2_fw** | AGTCTTACTCTCAAGGTCGATTGTGGTTCC |
| **Beauv_InFusion2_rev** | GAAACTGTCTGTGTAGCGCCTGTGATCGCACATCACC |
| **Beauv_InFusion3_fw** | TACACAGACAGTTTCTTGCTGCCTGTGCGATG |
| **Beauv_InFusion3_rev** | CGGCATCTACTGTTTTCACAAAGCCGAGTTTAGACTC |
| **Bass_InFusion1_fw** | CAGACATCACCGTTTACCATGGAGCCACCCAACAACGCCAATAC |
| **Bass_InFusion1_rev** | CCACAGGACCATCCAAAGCTAGCGCCGGGATGCTGGT |
| **Bass_InFusion2_fw** | TGGATGGTCCTGTGGAACAGTCATACTCGCAAGGT |
| **Bass_InFusion2_rev** | CACAAGGTCCGGATGGAGGTCATCTAGCTTGTTCG |
| **Bass_InFusion3_fw** | CATCCGGACCTTGTGATATTCAACTCGGTTCTTCAG |
| **Bass_InFusion3_rev** | CGGCATCTACTGTTTTCATAAAGACGCATTCAAAGCC |

Table S 3 - *A. niger* strains used in this study

| **Name** | **Relevant genotype** | **Source** |
| --- | --- | --- |
| **DSc1.4** | *pyrG^+^, prtT^-^, bbBeas* (AB1.13 derivative containing a single of pDS8.2 at *pyrG*) | this study |
| **DSc1.5** | *pyrG^+^*, *prtT^-^*, *bbBeas* (AB1.13 derivative containing a tandem copy of pDS8.2 at *pyrG*) | this study |
| **SB19.23** | *pyrG^+^*, *prtT^-^*, *bbBsls* (AB1.13 derivative containing a single of pSB22.3 at *pyrG*) | this study |
| **N402** | Wild type | [2] |
| **AB1.13** | *pyrG^-^*, *prtT^-^* | [3] |

Table S 4 - IC_50_ of tested compounds on parasites. Test strains used were *Trypanosoma brucei rhodesiense* (strain: STIB 900, stage: trypomastigotes), *Trypanosoma cruzi* (strain: Tulahuen C4, stage: amastigotes), *Leishmania donovani* (strain: MHOM-ET-67/L82, stage: amastigotes). Cytotoxicity was determined against rat skeletal muscle myoblast cells (L6 cell line). All values are given in µg/mL.

| **Compound** | ***T. b. rhod.*** | ***T. cruzi*** | ***L. donovani*** | ***Cytotox.*** |
| --- | --- | --- | --- | --- |
| Enniatin B | 0.670 | 0.475 | 0.460 | 2.63 |
| Bassianolide | 1.535 | 1.265 | 0.177 | 4.65 |
| Beauvericin | 1.277 | 0.599 | 0.244 | 1.52 |
| Bromo-beauvericin | 3.905 | >100 | 2.185 | >100 |
| Melarsoprol | 0.003 | n.d. | n.d | 11.4 |
| Benznidazole | n.d | 1.7 | n.d | >100 |
| Miltefosine | n.d | n.d | 0.235 | 56.1 |
| Podophyllotoxin | n.d | n.d | n.d | 0.013 |

Table S 5 - Quantification of beauvericin and bassianolide by MALDI-TOF. Defined concentrations of deuterated beauvericin or bassianolide (c_deut-beauv._ and c_deut-bass._) were mixed with varying concentrations of unlabelled beauvericin and bassianolide (c_beauv_. and c_bass._). The peak areas of the sodium adducts of the respective compounds (A_806.4_: unlabelled beauvericin, A_824.5_: labelled beauvericin, A_931.6_: unlabelled bassianolide, A_955.8_: labelled bassianolide) were determined and the ratios calculated.

| **Beauvericin** | | | | | |
| --- | --- | --- | --- | --- | --- |
| **A_806.4_** | **A_824.5_** | **A_806.4_/A_824.5_** | **c_beauv_. [µg/mL]** | **c_deut-beauv._ [µg/mL]** | **c_beauv_./_cdeut-beauv._** |
| 24001 | 2246 | 10.69 | 100 | 5 | 20 |
| 6328 | 928 | 6.75 | 50 | 5 | 10 |
| 935 | 179 | 5.22 | 25 | 5 | 5 |
| 1924 | 1395 | 1.38 | 10 | 5 | 2 |
| 14160 | 12734 | 1.11 | 5 | 5 | 1 |
| 12545 | 17574 | 0.71 | 2.5 | 5 | 0.5 |
| 450 | 3933 | 0.11 | 1 | 5 | 0.2 |
| **Bassianolide** | | | | | |
| **A_931.6_** | **A_955.8_** | **A_931.6_/A_955.8_** | **c_bass_. [µg/mL]** | **c_deut-bass._ [µg/mL]** | **c_bass_./_cdeut-bass._** |
| 10833 | 544 | 19.91 | 100 | 5 | 20 |
| 3503 | 291 | 12.04 | 50 | 5 | 10 |
| 8243 | 1742 | 4.73 | 25 | 5 | 5 |
| 9863 | 3332 | 2.96 | 10 | 5 | 2 |
| 6112 | 5281 | 1.16 | 5 | 5 | 1 |
| 769 | 1407 | 0.55 | 2.5 | 5 | 0.5 |
| 751 | 2759 | 0.27 | 1 | 5 | 0.2 |

**
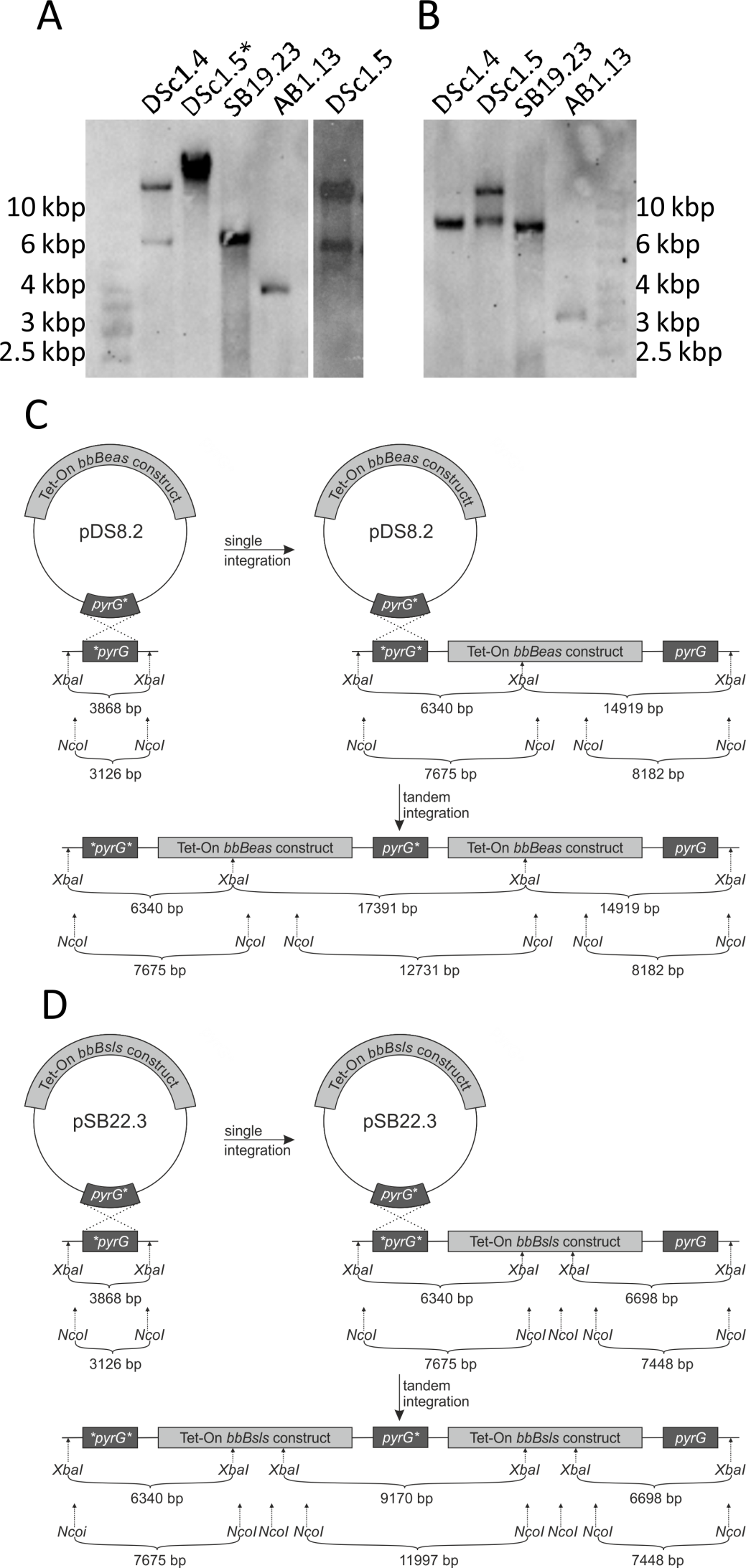
**


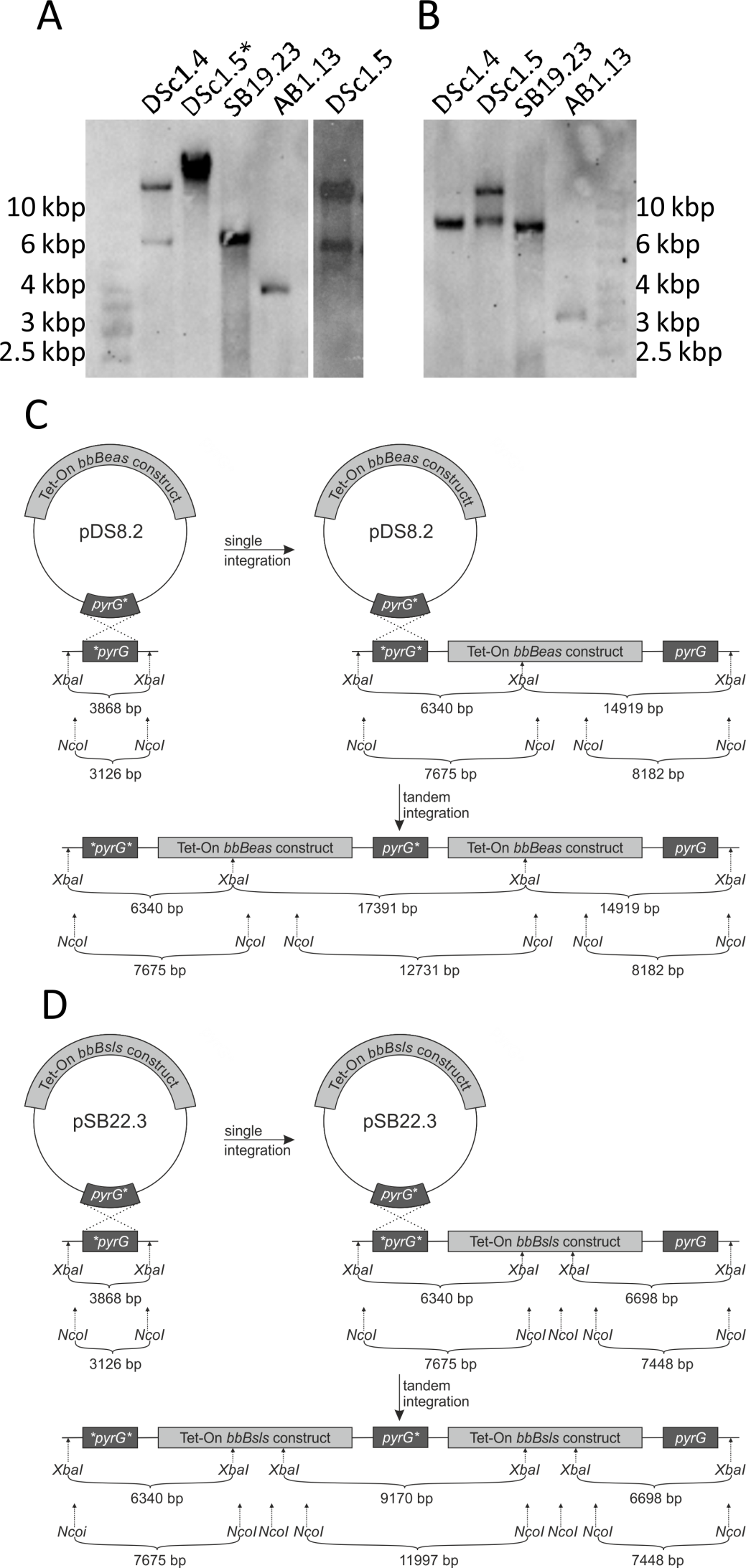


Figure S 1 - Southern blot analysis of transformants DSc1.4, DSc1.5 and SB19.23. A: *Xba*I restriction with *pyrG* as probe: DSc1.4: single integration of pDS8.2 at *pyrG* locus (6340 bp and 14919); DSc1.5*: incomplete restriction; SB19.23: single integration of pSB22.3 at *pyrG* locus (6340 bp and 6698 bp (both bands appear as a thick single band due to similar sizes); AB1.13: recipient strain (= no integration, 3868 bp); DSc1.5: tandem integration of pDS8.2 at *pyrG* locus (6340 bp, 14919 bp and 17391 bp). B: *Nco*I restriction with *pyrG* as probe: DSc1.4: single integration of pDS8.2 at *pyrG* locus (7675 bp and 8182 bp (both bands appear as a thick single band due to similar sizes); DSc1.5: tandem integration of pDS8.2 at *pyrG* locus (7675 bp, 8182 bp (appear as single band) and 12731); SB19.23: single integration of pSB22.3 at *pyrG* locus (7448 bp and 7675 bp (appear as single band)); AB1.13: recipient strain (= no integration, 3126 bp). C: Scheme of expected bands for no, single or tandem integration of pDS8.2 at the *pyrG* locus. D: Scheme of expected bands for no, single or tandem integration of pSB22.3 at the *pyrG* locus.


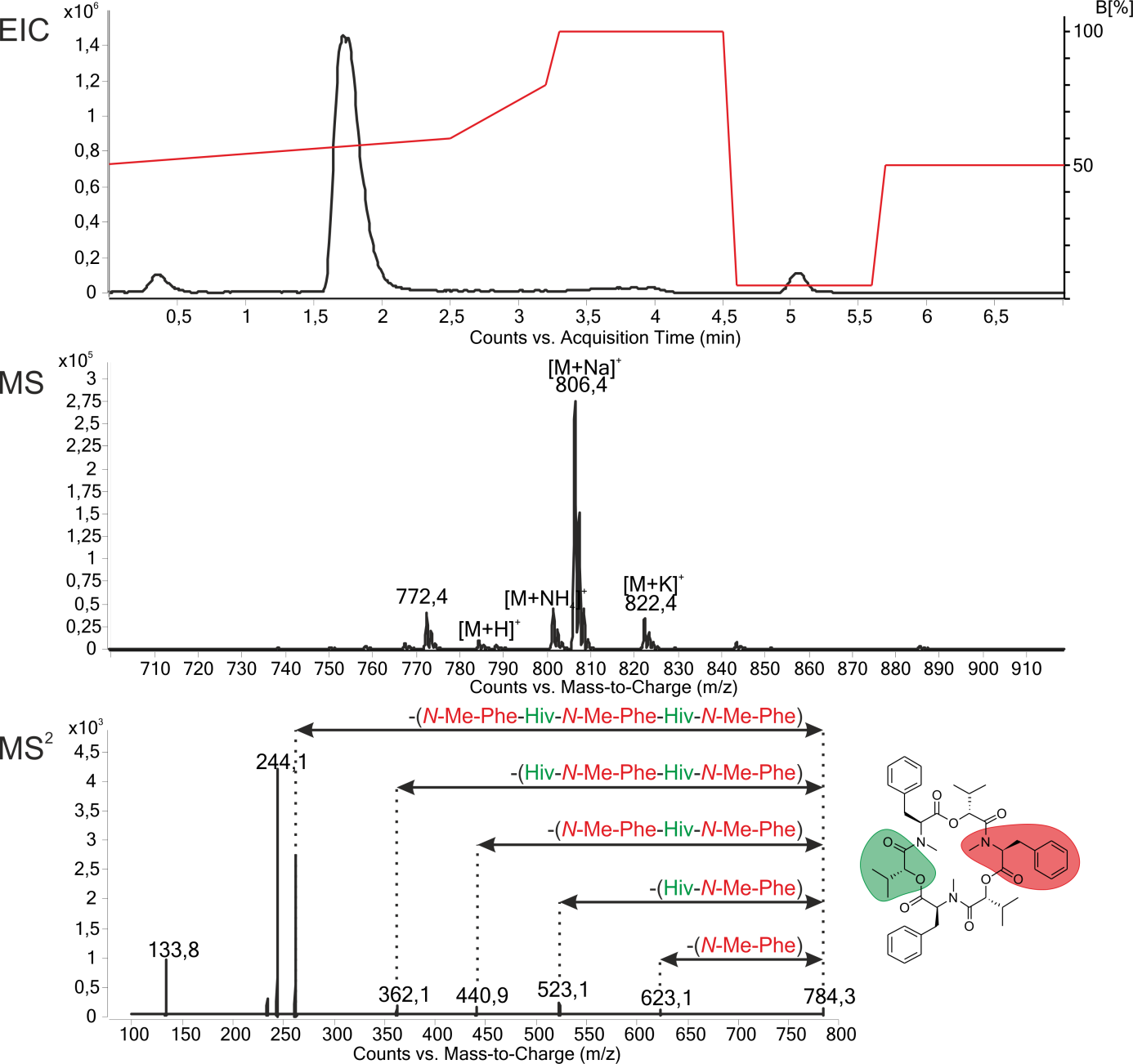


Figure S 2 - Analysis of heterologously produced beauvericin in *A. niger.* top: extracted ion chromatogram (EIC) of beauvericin extracted from DSc1.4. The solvent gradient is indicated by the red line. middle: mass spectrum of beauvericin. The H^+^, NH_4_^+^, Na^+^ and K^+^ adducts could be observed. bottom: Tandem mass spectrum of beauvericin. The peaks are assigned to the respective fragments. d-Hiv moieties are indicated in green, *N*-Me-l-Phe moieties in red.


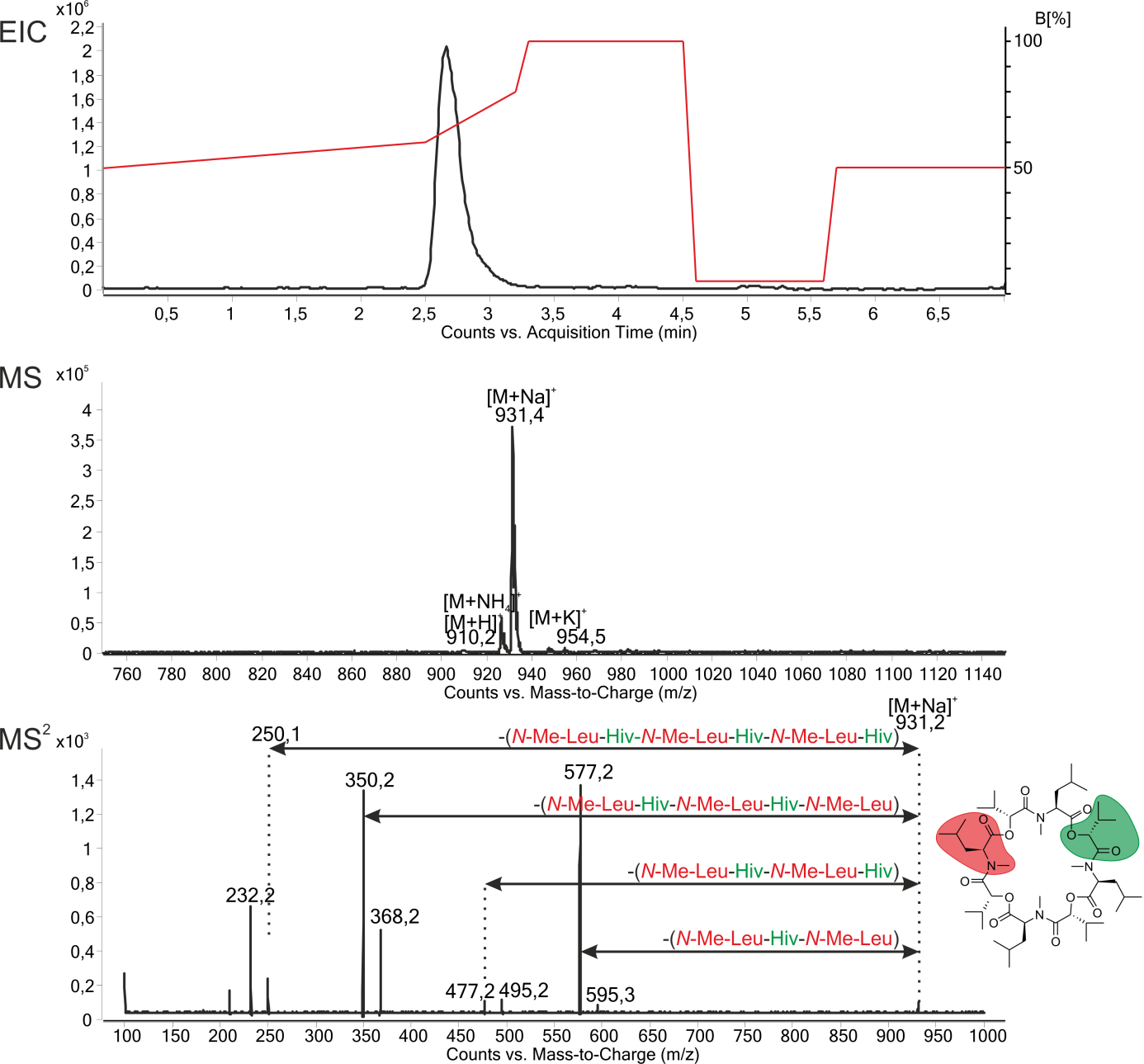


Figure S 3 - Analysis of heterologously produced bassianolide in *A. niger*. top: extracted ion chromatogram (EIC) of bassianolide extracted from SB19.23. The solvent gradient is indicated by the red line. middle: mass spectrum of bassianolide. The H^+^, NH_4_^+^, Na^+^ and K^+^ adducts could be observed. bottom: Tandem mass spectrum of bassianolide. The peaks are assigned to the respective fragments. d-Hiv moieties are indicated in green, *N*-Me-l-Leu moieties in red.


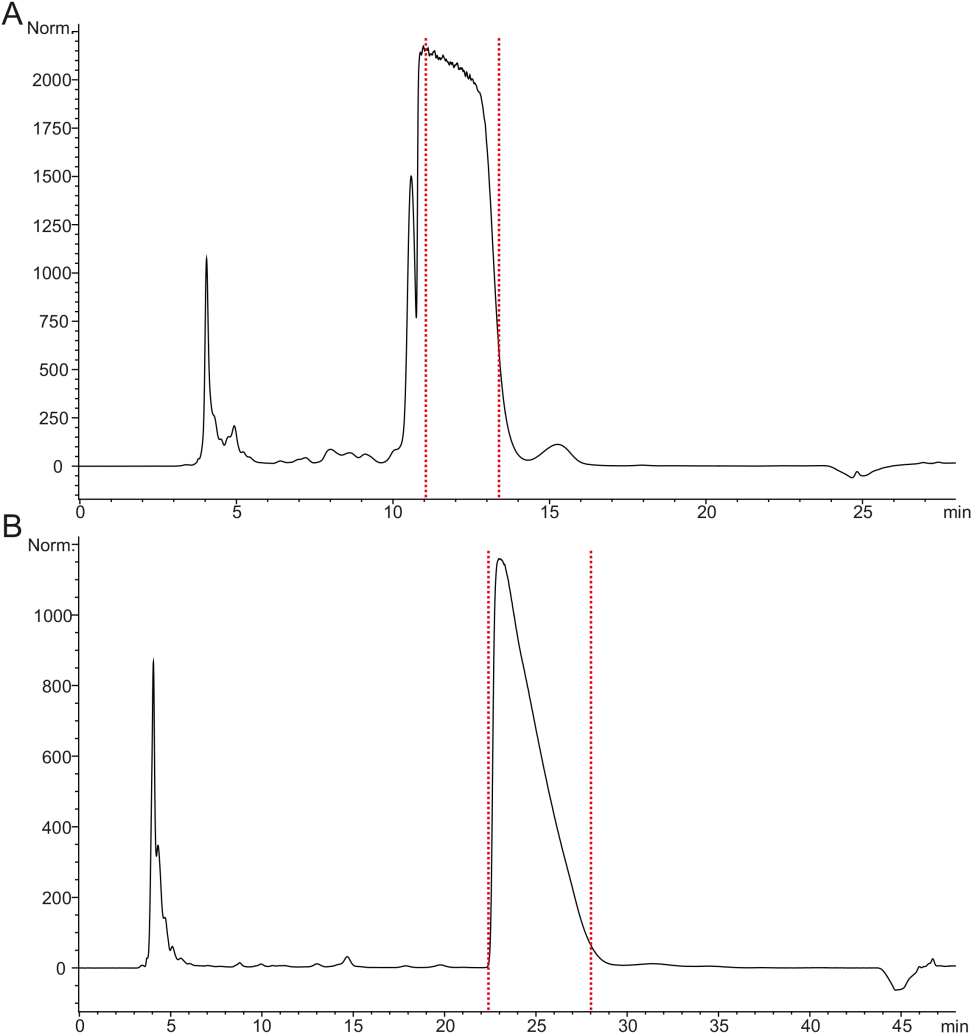


Figure S 4 - Preparative HPLC chromatogram of CDP purification. A: Chromatogram of purification of beauvericin (overloaded), B: Chromatogram of purification of bassianolide. Chromatogram was recorded with a multiple wavelength recorder (λ = 215 nm shown). The dashed red lines indicated the fractions that were pooled and contained only the respective CDP without any detectable impurities.


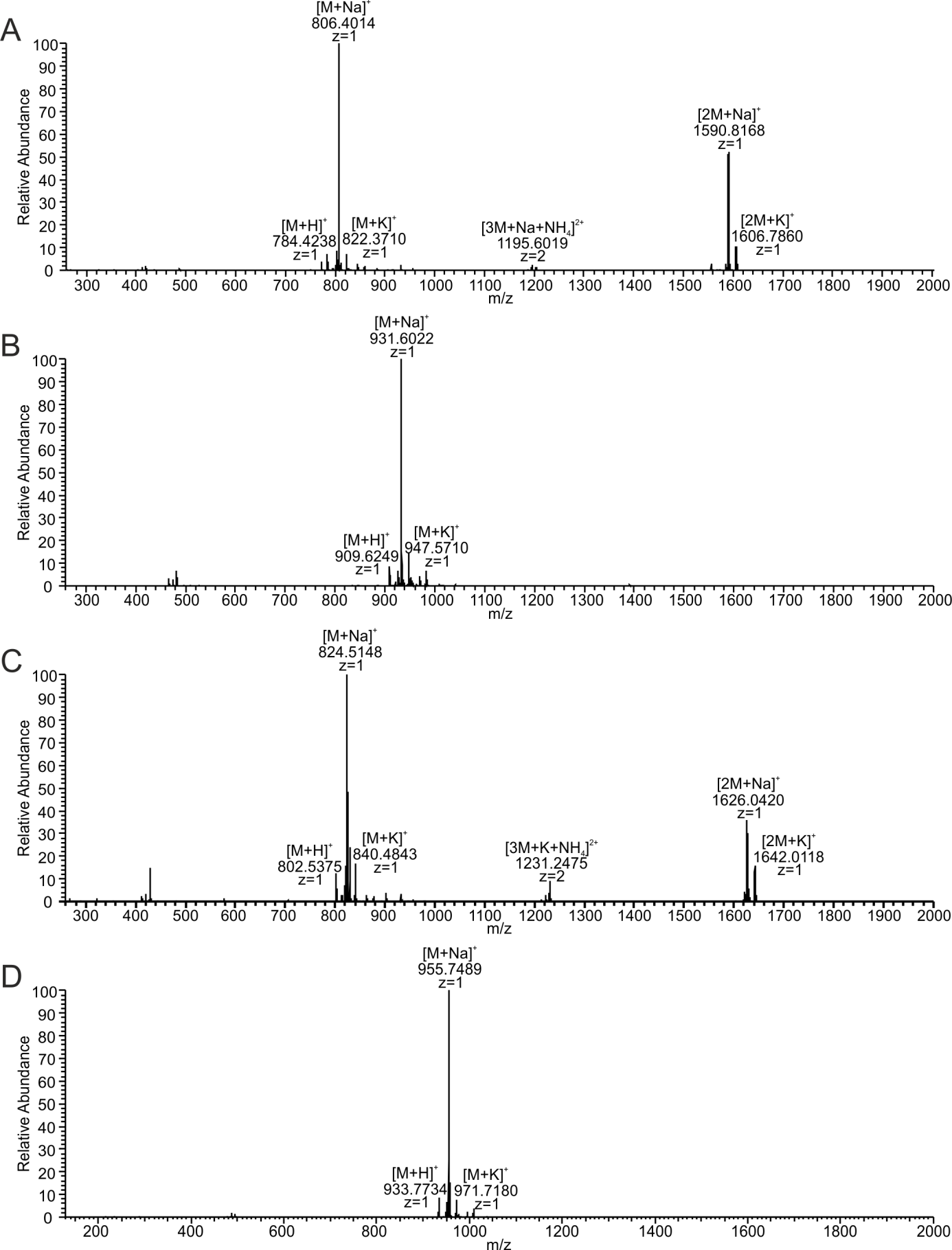


Figure S 5 - Mass spectra of purified beauvericin and bassianolide. A: Spectrum of purified beauvericin; Next to the monomer of beauvericin, dimers and trimers could be detected. B: Spectrum of purified bassianolide. C: Spectrum of purified beauvericin-d_18_; Next to the monomer of beauvericin-d_18_, dimers and trimers could be detected. D: Spectrum of purified bassianolide-d_24_.The purified compounds were solved in methanol and MS-spectra were recorded by direct injection of the solutions.


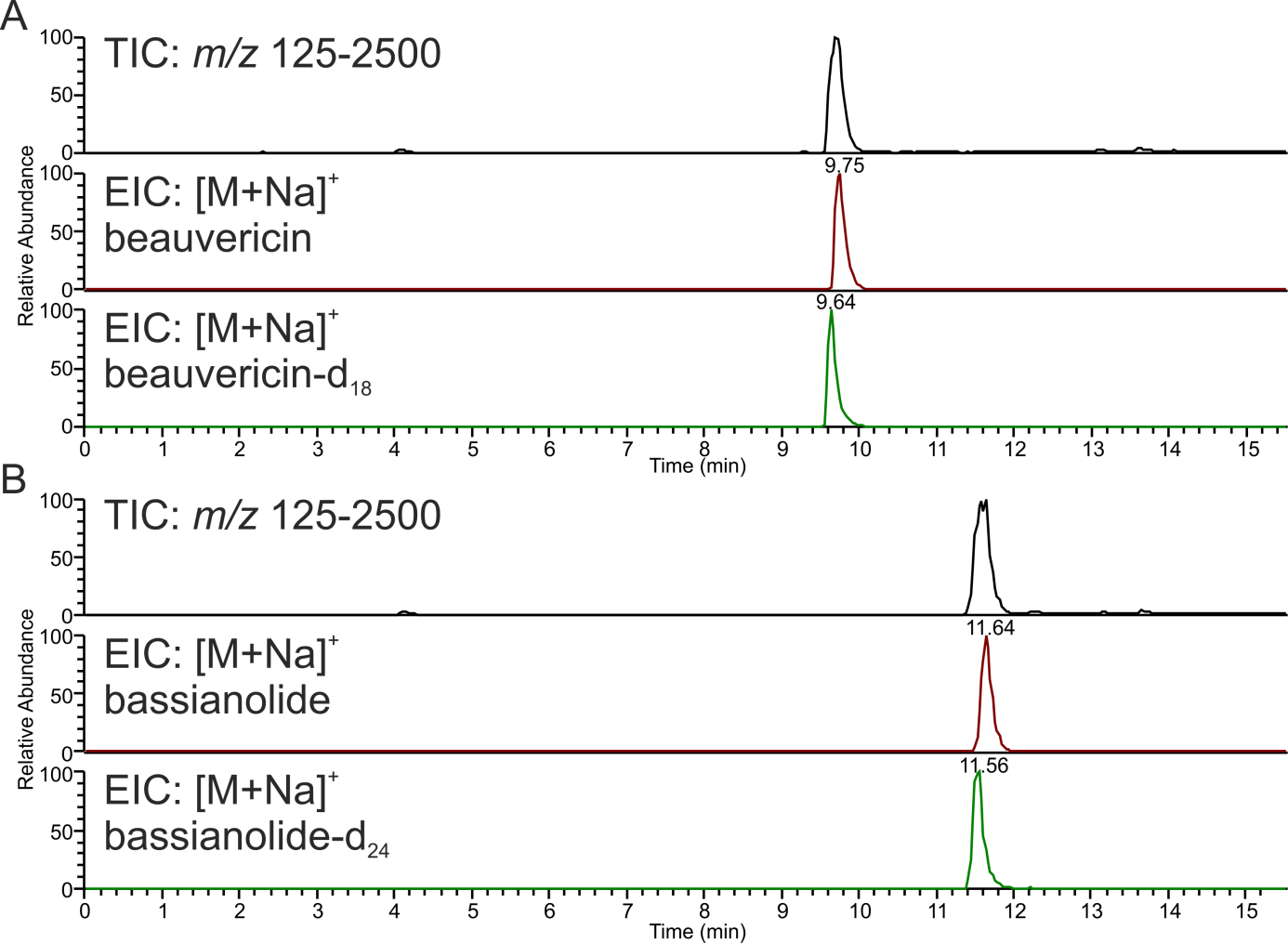


Figure S 6 - Retention times of labeled and unlabeled metabolites. A: Retention times of beauvericin (Rt = 9.75 min) and beauvericin-d_18_ (Rt = 9.64 min), B: Retention times of bassianolide (Rt = 11.64 min) and bassianolide-d_24_ (Rt = 11.56 min). TIC: total ion chromatogram, EIC: extracted ion chromatogram of the [M+Na]^+^ adduct of the respective metabolite.

**Beauvericin**: ^1^H NMR (700 MHz, CDCl_3_): δ 7.19-7.29 (15H, m), 5.61 (3H, d, *J* = 8.4 Hz), 4.85 (3H, d, *J* = 8.5 Hz), 3.42 (3H, dd, *J* = 14.8 Hz, 4.9 Hz), 3.05 (9H, s), 2.96 (3H, dd, *J* = 14.7 Hz, 12.2Hz), 1.93-2.00 (3H, m), 0.82 (9H, d, *J* = 6.6 Hz), 0.39 (9H, d, *J* = 6.8 Hz); ESI-HRMS: *m/z* calculated for [C_45_H_57_N_3_NaO_9_]^+^: 806.3987, found: 806.4014.

**Bassianolide**: ^1^H NMR (700 MHz, CDCl_3_): δ 4.43-5.67 (8H, m), 2.86-3.25 (12H, m), 0.55-2.27 (72H, m); ESI-HRMS: *m/z* calculated for [C_48_H_84_N_4_NaO_12_]^+^: 931.5978, found: 931.6022.


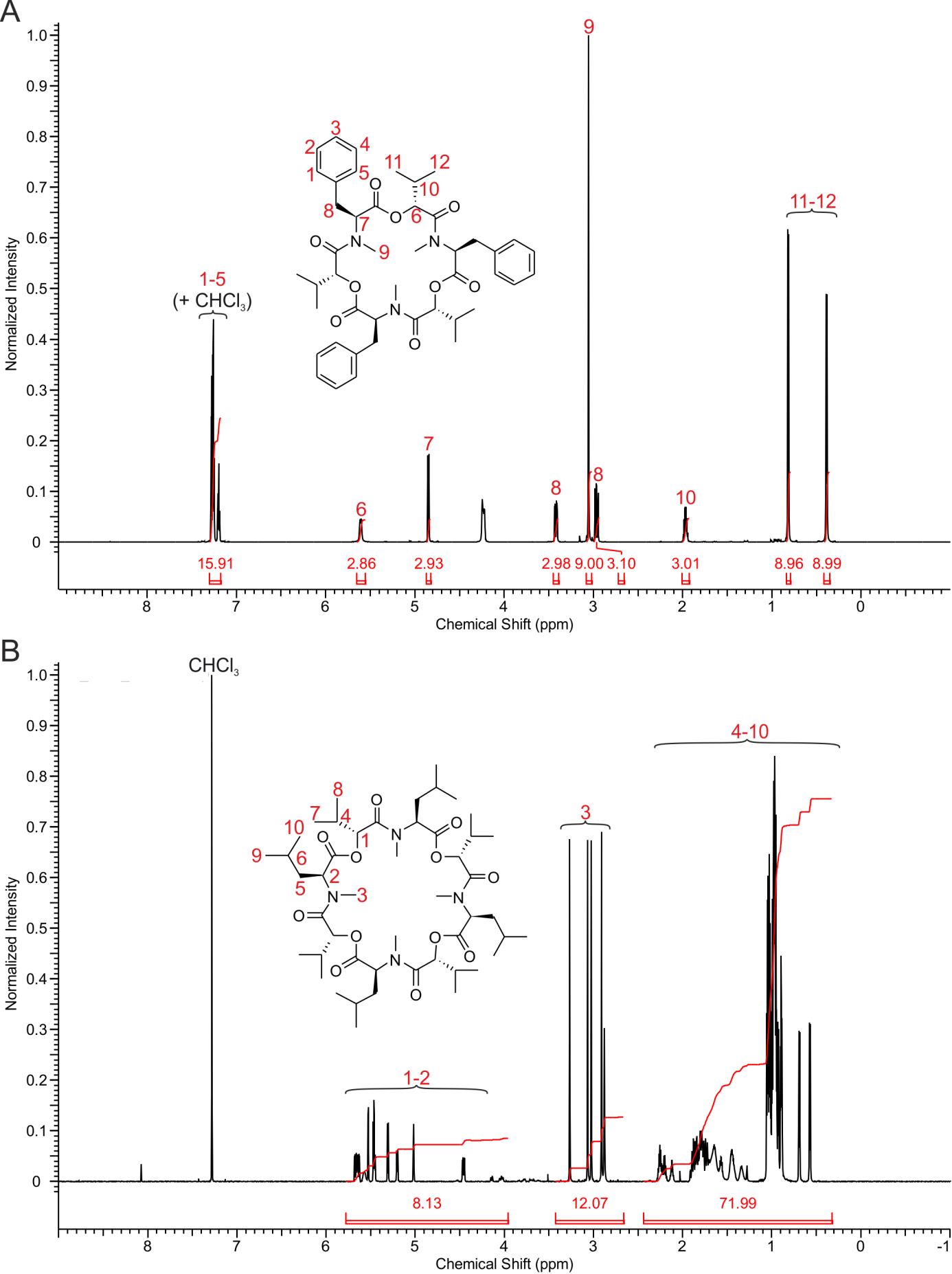


Figure S 7 - ^1^H-NMR spectra of purified beauvericin and bassianolide in CDCl_3_. A: Spectrum of purified beauvericin; ^1^H proton signals could be assigned to the corresponding protons; integrals correspond to number of ^1^H atoms in molecule. B: Spectrum of purified bassianolide, due to different conformations, ^1^H atoms could be assigned to corresponding regions in the spectrum; integrals correspond to the number of ^1^H atoms in molecule.

For beauvericin, all signals can be assigned to the corresponding ^1^H atoms (**A**) and the chemical shifts match the data from the literature [4]. Although bassianolide is, as beauvericin, a highly symmetrical molecule, the ^1^H-NMR spectrum is more complex, due to different conformational populations it can adopt. Therefore, for example, the H atoms of the *N*-methyl group do not give a single signal as for beauvericin but are split into five individual signals reflecting two different conformations (one symmetrical conformation giving one signal and one asymmetrical conformation giving four signals) (δ 2.86 - 3.25 ppm, **B**). The same effect was observed for all other signals as well, impeding an exact assignment of the peaks to the corresponding H atoms due to overlaid signals. However, integration and normalization of the signal intensities of the five *N*-methyl groups to the corresponding twelve H atoms leads to an overall integral which exactly matches the expected number of proton intensities (**B**).


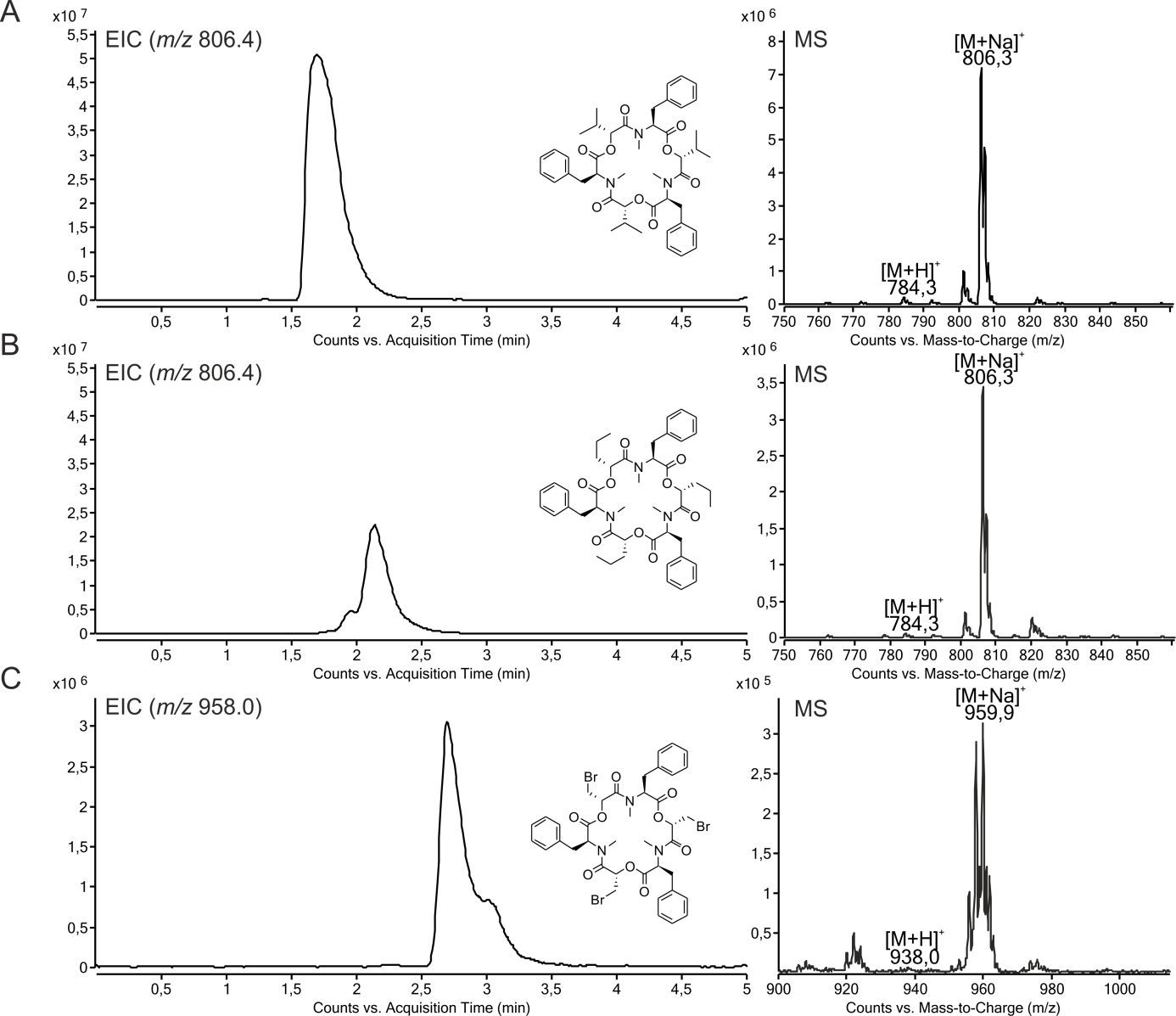


Figure S 8 - Extracted ion chromatograms of beauvericin analogues produced in *A. niger*. A: EIC (*m/z* 806.4, [M+Na]^+^) and mass spectrum of natural beauvericin. B: EIC (*m/z* 806.4, [M+Na]^+^) and mass spectrum of 2-hydroxyvalerate-beauvericin (4). C: EIC (*m/z* 958.0, [M+Na]^+^) and mass spectrum of bromo-beauvericin (1).


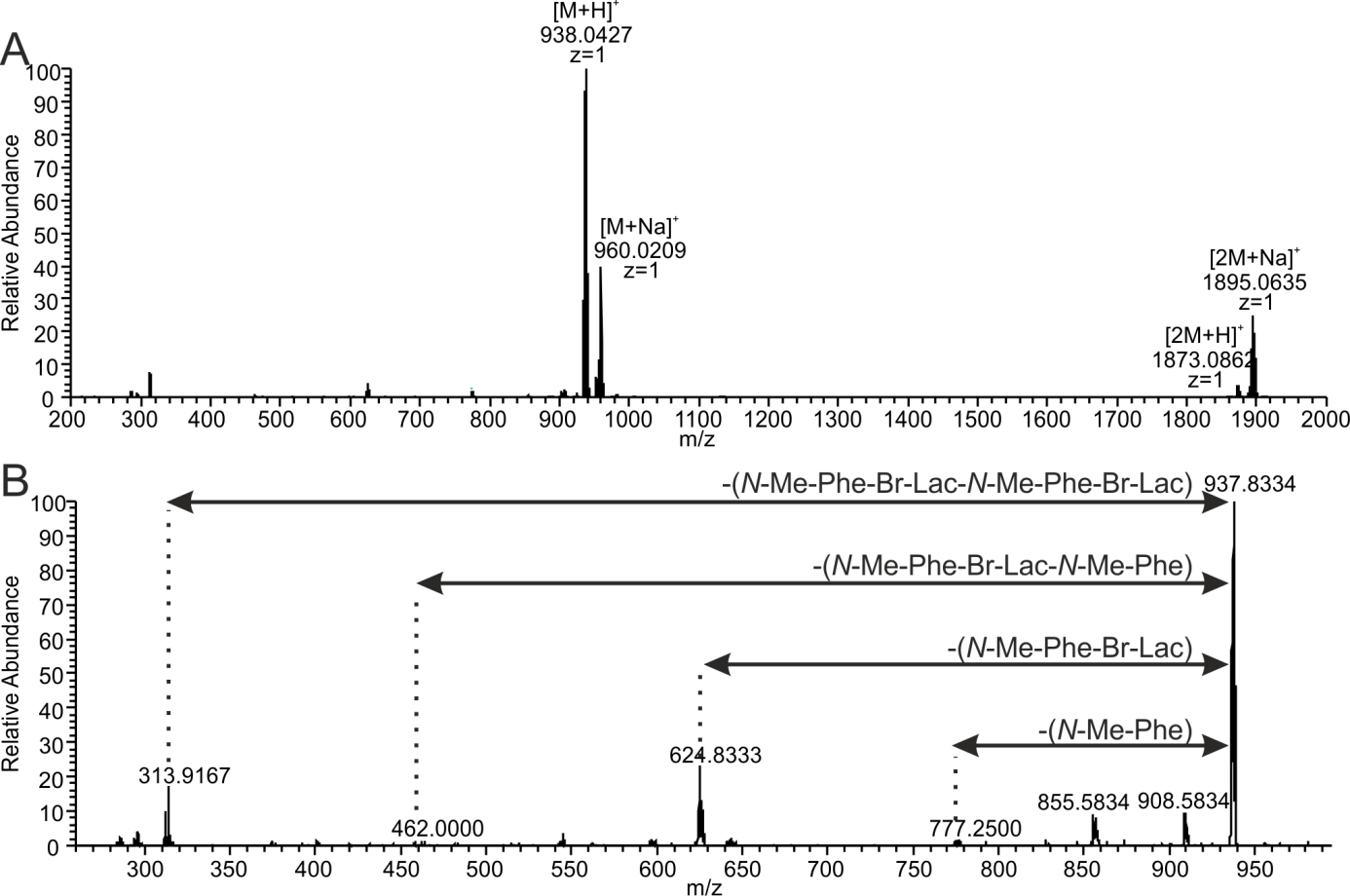


Figure S 9 - (Tandem-)Mass spectrum of purified bromo-beauvericin. A: MS of purified bromo-beauvericin; next to the monomer, dimers could be detected. B: MS^2^ of purified bromo-beauvericin. The peaks are assigned to the respective fragment. *N*-Me-Phe: *N*-methylphenylalanine, Br-Lac: 3-bromolactate.

**Bromo-beauvericin**: ^1^H NMR (400 MHz, D_3_COD): δ 5.65-5.78 (15H, m), 4.13 (3H, dd, *J* = 7.3 Hz, 6.4 Hz), 3.67 (3H, d, *J* = 10.3 Hz, 5.3 Hz), 1.73-1.88 (9H, m), 1.55 (3H, dd, *J* = 14.2 Hz, 10.6 Hz), 1.49 ppm (9H, s); ^13^C NMR (100.6 MHz, D_3_COD): δ 27.61, 32.16, 33.92, 58.50, 69.28, 126.48, 128.20, 128.90, 136.42, 167.20, 168.83 ppm; ESI-HRMS: *m/z* calculated for [C_39_H_43_Br_3_N_3_O_9_]^+^: 936.0523, found: 936.0513.


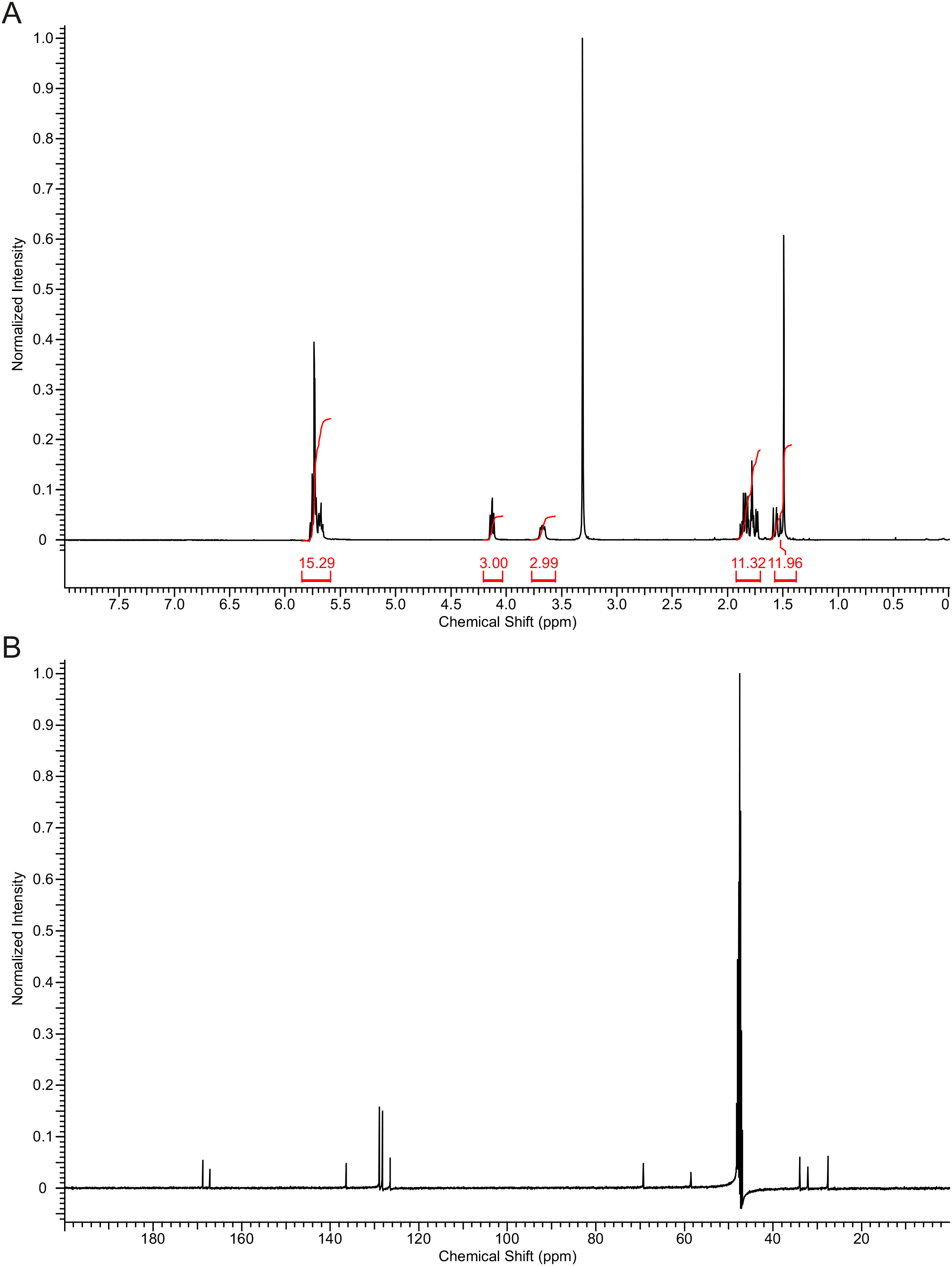


Figure S 10 - ^1^H-NMR and ^13^C-NMR spectra of purified bromo-beauvericin in MeOH-d_4_. A: ^1^H-NMR spectrum of purified bromo-beauvericin; B: ^13^C-NMR spectrum of purified bromo-beauvericin.


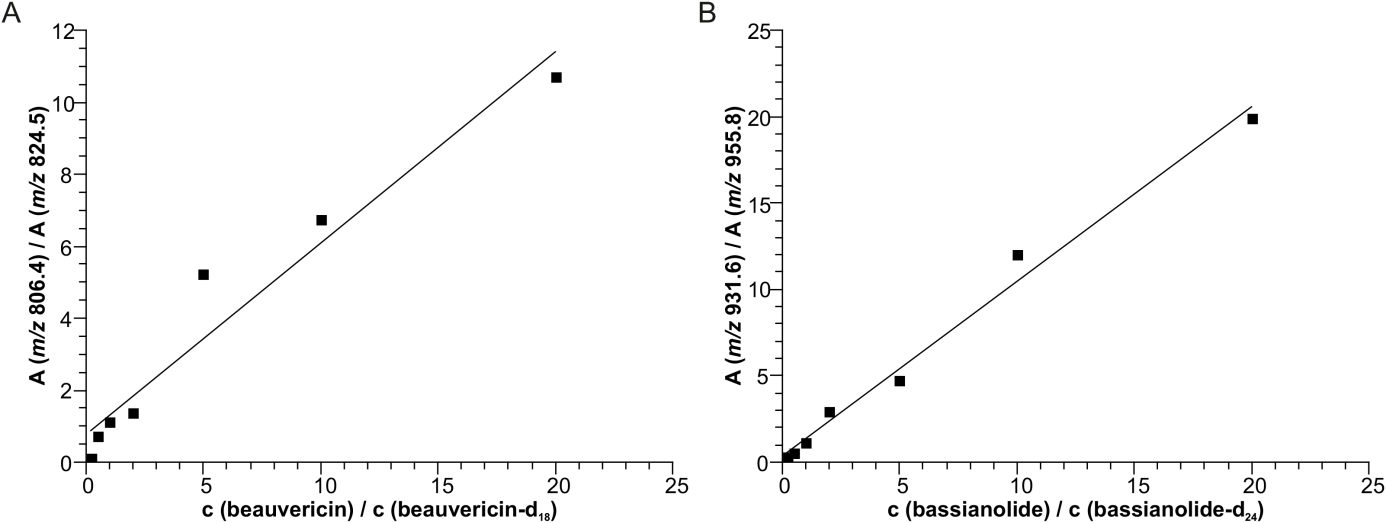


Figure S 11- Correlation between ratio of peak areas and concentration of labelled and unlabelled compounds. A linear correlation between the ratio of the peak areas of the sodium adduct of labeled and unlabeled beauvericin (A) and bassianolide (B) and the ratio of the concentration of the respective labeled and unlabeled compounds can be observed. The coefficient of determination is for plot A *R*^2^ = 0.946 and for plot B *R*^2^ = 0.988.

**References**

1. Meyer V, Wanka F, van Gent J, *et al*. (2011) Fungal gene expression on demand: an inducible, tuneable, and metabolism-independent expression system for *Aspergillus niger*. Appl Environ Microbiol 77:2975–2983. doi: 10.1128/AEM.02740-10

2. Bos CJ, Debets AJM, Swart K, *et al*. (1988) Genetic analysis and the construction of master strains for assignment of genes to six linkage groups in *Aspergillus niger*. Curr Genet 14:437–443.

3. Mattern IE, van Noort JM, van den Berg P, *et al*. (1992) Isolation and characterization of mutants of *Aspergillus niger* deficient in extracellular proteases. Mol Gen Genet 234:332–336.

4. Zhan J, Burns AM, Liu MX, *et al*. (2007) Search for cell motility and angiogenesis inhibitors with potential anticancer activity: beauvericin and other constituents of two endophytic strains of *Fusarium oxysporum*. J Nat Prod 70:227–232. doi: 10.1021/np060394t
